# Synthesis of Aromatic Aza-metallapentalenes from Metallabenzene via Sequential Ring Contraction/Annulation

**DOI:** 10.1038/srep09584

**Published:** 2015-04-09

**Authors:** Tongdao Wang, Feifei Han, Haiping Huang, Jinhua Li, Hong Zhang, Jun Zhu, Zhenyang Lin, Haiping Xia

**Affiliations:** 1 State Key Laboratory of Physical Chemistry of Solid Surfaces and Collaborative Innovation Center of Chemistry for Energy Materials (iChEM), and Department of Chemistry, Xiamen University, Xiamen 361005, China; 2 Department of Chemistry, The Hong Kong University of Science and Technology, Clear Water Bay, Kowloon, Hong Kong

## Abstract

The concept of aromaticity has long played an important role in chemistry and continues to fascinate both experimentalists and theoreticians. Among the archetypal aromatic compounds, heteroaromatics are particularly attractive. Recently, substitution of a transition-metal fragment for a carbon atom in the anti-aromatic hydrocarbon pentalene has led to the new heteroaromatic osmapentalenes. However, construction of the aza-homolog of osmapentalenes cannot be accomplished by a similar synthetic manipulation. Here, we report the synthesis of aza-osmapentalenes by sequential ring contraction/annulation reactions of osmabenzenes via osmapentafulvenes. Nuclear magnetic resonance spectra, X-ray crystallographic analysis, and DFT calculations all suggest that these aza-osmapentalenes exhibit aromatic character. Thus, the stepwise transformation of metallabenzenes to metallapentafulvenes and then aza-metallapentalenes provides an efficient and facile synthetic route to these bicyclic heteroaromatics.

As one of the most fundamental chemistry concepts, aromaticity has been continually investigated since Faraday's discovery of benzene in 1825^1^ and Kekulé's famous alternating single- and double-bond cyclic structure proposed in 1865^2^,^3^. The Hückel aromaticity rule^4^ is applied to cyclic compounds containing 4n + 2π electrons, but Möbius topologies favour 4n delocalized π electron counts^5^,^6^,^7^,^8^. In general, aromatic compounds are substantially more stable thermodynamically and anti-aromatic compounds less stable than appropriate non-aromatic reference systems. Synergistic interplay of theoretical and experimental investigations has led to the synthesis and characterisation of numerous aromatic molecules with interesting structural features and properties^9^,^10^. The incorporation of heteroatoms or related groups into aromatic hydrocarbons can result in various heteroaromatic compounds. These introduced heteroatoms are not limited to main-group atoms or related groups; they also include transition-metal fragments. As a result of the increasing structural and compositional variety of aromatic species, the definitions and criteria for characterising aromaticity have also been developing rapidly^11^.

Metallaaromatics^12^,^13^,^14^ are organometallic species derived from the replacement of a (hydro)carbon unit in conventional aromatic hydrocarbons with a transition-metal fragment. As typical metallaaromatic compounds, metallabenzene complexes have been extensively studied by both experimentalists and theoreticians, dating back to the first computationally proposed metallabenzene complexes by Thorn and Hoffman^15^ in 1979. The increase in the variety of the metallaaromatic family is further evidenced by the recent discovery of metallapyridines^16^,^17^,^18^, which are azaheterocyclic analogues of metallabenzenes. Very recently, efforts to expand the aromatic family has also led to the discovery of the first metallapentalene (osmapentalene)^19^,^20^, which has been shown to exhibit aromaticity. Theoretical computations reveal that all these osmapentalenes with planar metallacycle benefit from Craig-Möbius aromaticity. The involvement of transition-metal *d* orbitals in the π conjugation switches the Hückel anti-aromaticity of pentalene into the Möbius aromaticity of metallapentalene. The introduction of nitrogen atoms into the pentalene system lead to the heterocyclic analogs of pentalene which are broadly defined as azapentalenes. Similar to the parent pentalenes, azapentalenes are anti-aromatic by virtue of a 8-π-electron system^21^. Some azapentalene molecules that contain nitrogen atoms in the bridgehead positions can be considered as analogs of a pentalene dianion, and these molecules are actually aromatic^22^. The isolation of the metallapentalene prompted us to investigate whether an aza-metallapentalene exists and, if so, how the synthesis could be achieved. As shown in Fig. 1, the structure of an aza-metallapentalene resembles that of pentalene, which is generally considered to be an anti-aromatic molecule, prompting us to consider whether an aza-metallapentalene, which is structurally and electronically analogous to metallapentalene, is also aromatic. In this paper, we present the synthesis and characterisation of aza-metallapentalenes, which were obtained from metallabenzenes via sequential ring contraction/annulation reactions.

## Results

### Synthesis of the first aza-metallapentalene

**Figure 2:**
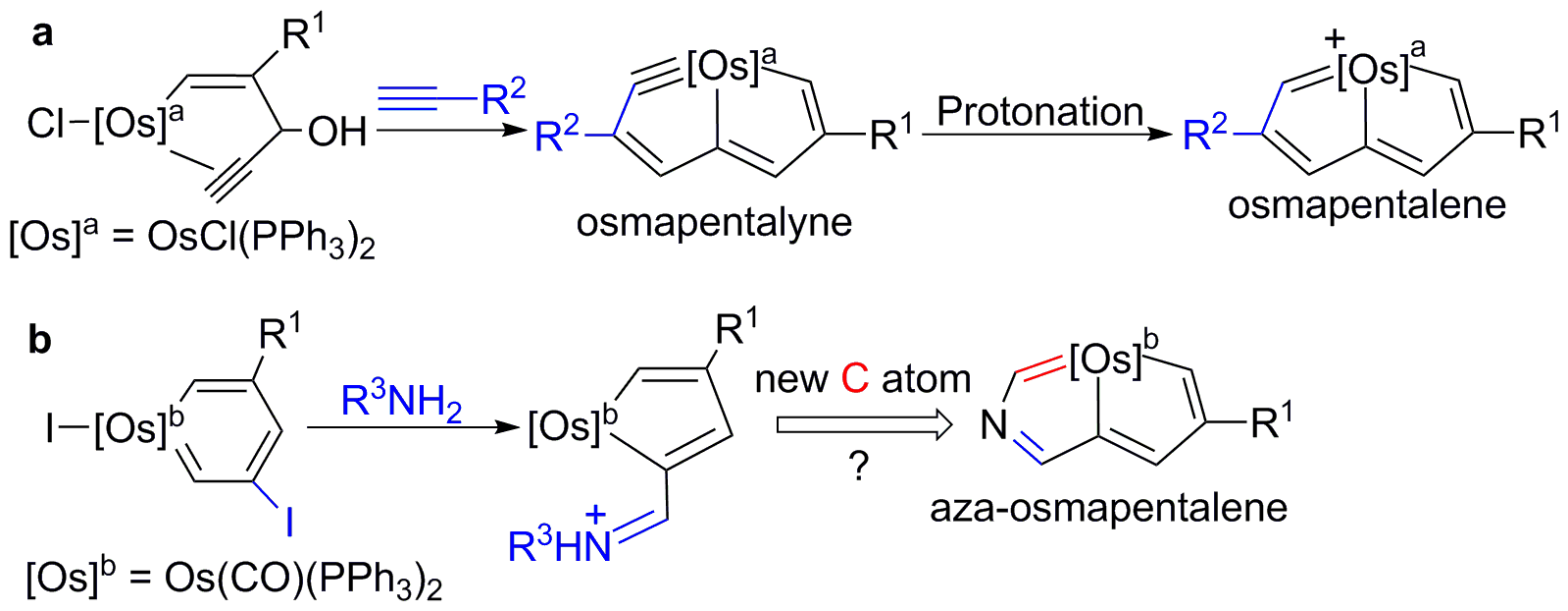
Previous work and initial attempt. (a) Protonation of osmapentalyne produces aromatic osmapentalene. (b) Reactions of osmabenzene with amines, leading to the formation of osmacyclopentadiene with an exocyclic C = N bond.

The recently reported osmapentalene^19^,^20^ was synthesised (derived) from the protonation of osmapentalyne (Fig. 2a). Experimentally, osmapentalyne^23^ was observed to be thermally stable and easy to prepare via the reaction of osmium complexes with terminal alkynes (Fig. 2a). Inspired by this observation, we initially attempted to react osmium complexes with a number of nitriles such as benzonitrile or acetonitrile in the hope of obtaining the corresponding aza-osmapentalynes. However, the expected reactions did not occur, only osmabenzenes or other osmacycles were detected, which have been previously reported^24^,^25^. Therefore, the preparation of an aza-metallapentalene via the synthesis of an aza-osmapentalyne followed by protonation is unfeasible.

Previously, we synthesised osmacyclopentadienes, each bearing an exocyclic C = N bond, by reacting an osmabenzene complex with amines^26^ (Fig. 2b). We envisioned that an aza-osmapentalene might be directly obtained by introducing a new carbon atom, with the intention of inducing a further annulation reaction yielding an osmacyclopentadiene monocyclic system, as illustrated by the retrosynthetic analysis shown in Fig. 2b.

**Figure 3:**
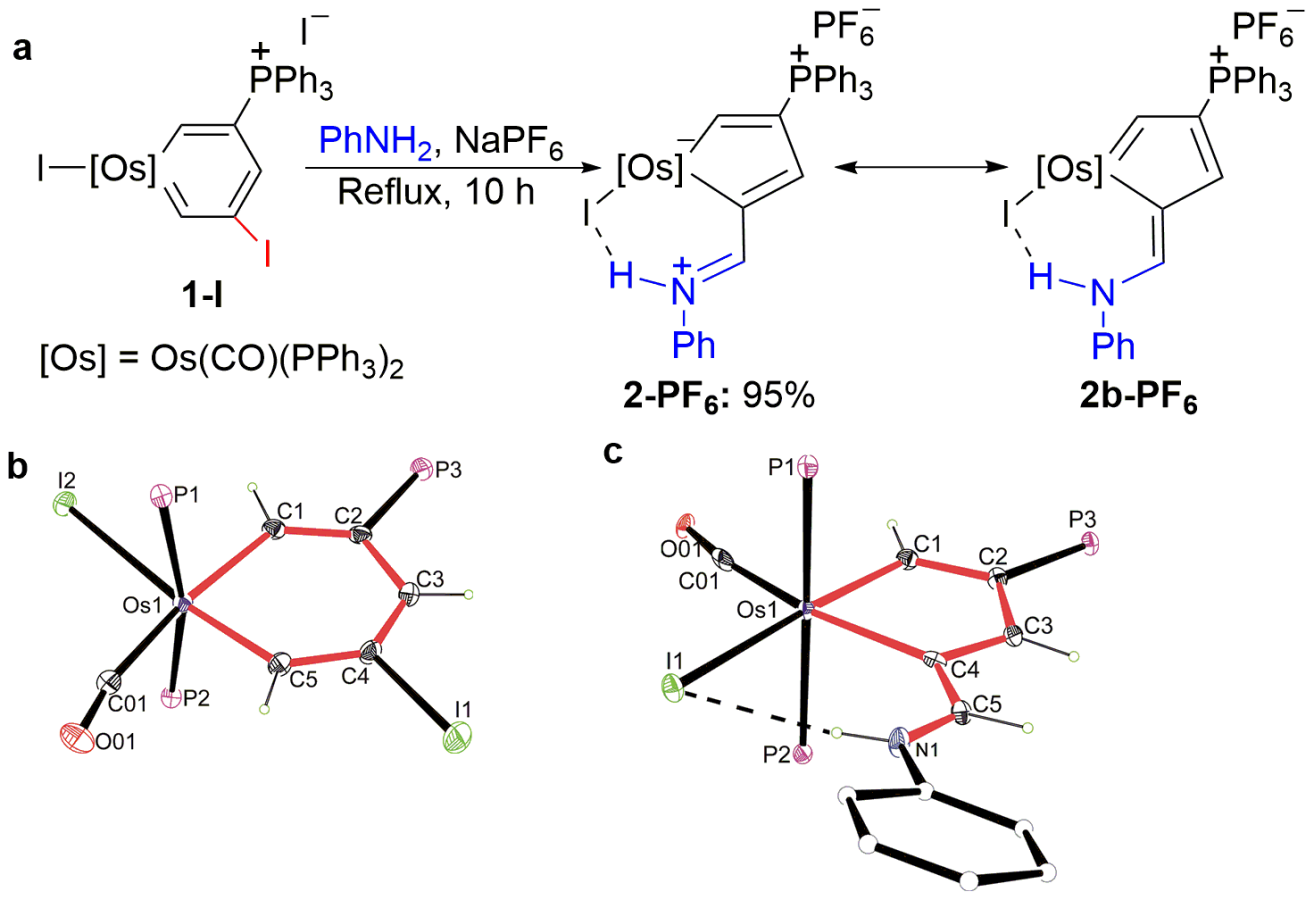
Synthesis of osmacyclopentadiene 2-PF_6_. (a) The reaction of osmabenzene **1-I** with aniline produces osmacyclopentadiene **2-PF_6_**. (b, c) X-ray structures of **1-I** (b) and **2-PF_6_** (c) (50% probability level). Phenyl moieties in PPh_3_, the counter anion and the solvent molecules have been omitted for clarity. Selected bond lengths (Å) for **1-I**: Os1-C1 2.065(7), Os1-C5 1.956(7), C1-C2 1.359(10), C2-C3 1.432(11), C3-C4 1.345(10), C4-C5 1.407(11), C4-I1 2.131(7), Os1-C01 1.925(8), C01-O01 1.145(9), Os1-I2 2.8161(8). Selected bond lengths (Å) for **2-PF_6_**: Os1-C1 2.004(6), Os1-C4 2.183(7), Os1-I1 2.8347(5), C1-C2 1.372(9), C2-C3 1.430(9), C3-C4 1.386(9), C4-C5 1.418(9), C5-N1 1.309(8), Os1-C01 1.880(9), C01-O01 1.109(8).

We first modified an osmabenzene complex by ligand substitution to generate the more stable osmabenzene **1-I**. The structure of osmabenzene **1-I** was verified by X-ray diffraction analysis (Fig. 3b; the relevant experimental details are presented in Supplementary Figures and Methods). Subsequent treatment of **1-I** with aniline in the presence of sodium hexafluorophosphate afforded the expected osmacyclopentadiene **2-PF_6_** in 95% yield (Fig. 3a). The complex **2-PF_6_** was characterised by multinuclear nuclear magnetic resonance (NMR) spectroscopy, single-crystal X-ray diffraction analysis, and high-resolution mass spectroscopy. The structure of **2-PF_6_** is shown in Fig. 3c. The X-ray diffraction analysis demonstrated that **2-PF_6_** contains a coplanar metallacyclopentadiene ring with an exocyclic imino group. The structural parameters indicated that the metallacycle of **2-PF_6_** could be represented by two resonance structures, **2-PF_6_** and **2b-PF_6_** (Fig. 3a), with **2-PF_6_** as the more dominant structure. The structural data (averaged bond lengths) also indicated that both the metallacycles in **1-I** and **2-PF_6_** have delocalised structures. In addition, the six atoms Os1 and C1-C5 in each complex are approximately coplanar, which is reflected by their small mean deviation (0.0129 Å for **1-I**; 0.0331 Å for **2-PF_6_**) from the least-squares plane.

As shown in Fig. 3a, the resonance structure **2b-PF_6_** (metallapentafulvene) contributes to the overall structure of the complex **2-PF_6_**. Since pentafulvene molecules^27^ are normally considered aromatic, we assume that the metallapentafulvene **2-PF_6_** is also aromatic, as is indeed supported by our density functional theory (DFT) results (*vide infra*).

**Figure 4:**
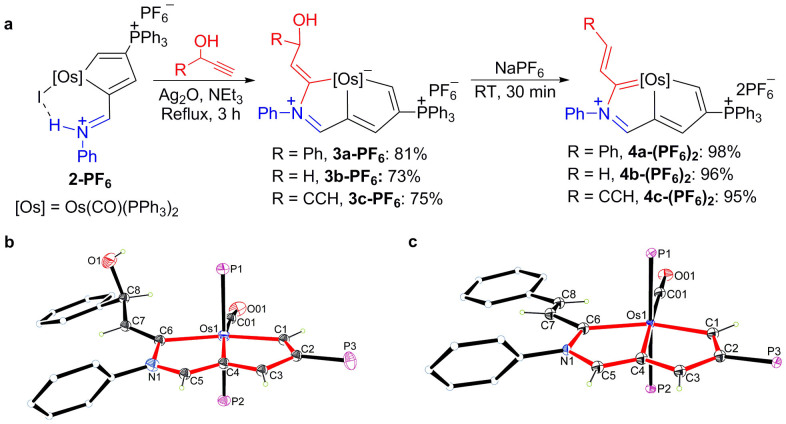
Synthesis of aza-osmapentalene 4-(PF_6_)_2_. (a) The reaction of osmacyclopentadiene **2-PF_6_** with substituted propynols followed by dehydroxylation produces aza-osmapentalene **4-(PF_6_)_2_**. (b, c) X-ray structures of **3a-PF_6_** (b) and **4a-(PF_6_)_2_** (c) (50% probability level). Phenyl moieties in PPh_3_, the counter anion and the solvent molecules have been omitted for clarity. Selected bond lengths (Å) for **3a-PF_6_**: Os1-C1 2.040(8), Os1-C4 2.100(7), Os1-C6 2.180(9), C1-C2 1.381(11), C2-C3 1.450(11), C3-C4 1.373(12), C4-C5 1.377(13), C5-N1 1.313(11), C6-N1 1.483(11), C6-C7 1.338(12), C7-C8 1.489(12), C8-O1 1.430(12). Selected bond lengths (Å) for **4a-(PF_6_)_2_**: Os1-C1 2.041(4), Os1-C4 2.069(4), Os1-C6 2.082(4), C1-C2 1.407(5), C2-C3 1.410(5), C3-C4 1.394(5), C4-C5 1.381(5), C5-N1 1.374(5), C6-N1 1.396(5), C6-C7 1.465(5), C7-C8 1.337(5).

According to the retrosynthetic analysis shown in Fig. 2b, the complex **2-PF_6_** could be used as the precursor to afford the desired aza-metallapentalene by introducing an additional carbon atom. To test this idea, reactions of **2-PF_6_** with the propynols RCH(OH)C ≡ CH in the presence of Ag_2_O were carried out in the hope of obtaining the desired aza-osmapentalene complexes. Triethylamine was used in the reactions to ensure that an acetylide ligand could be easily delivered to the osmium metal centre (Warning: the reaction may proceed through the formation of silver acetylide, which decomposes violently on contact with moisture and water producing highly flammable and explosive acetylene gas, and causing fire and explosion hazard). As shown in Fig. 4a, treatment of **2-PF_6_** with phenylpropynol in the presence of Ag_2_O and triethylamine under reflux for approximately 3 h led to the formation of complex **3a-PF_6_**, which was isolated in 81% yield. The complex **3a-PF_6_** was characterised by NMR spectroscopy and HRMS, and its structure was determined by X-ray crystal structure analysis.

Complex **3a-PF_6_** exhibits an essentially planar metal-bridged bicyclic structure (Fig. 4b). The mean deviation from the least-squares plane through Os1, N1 and C1-C6 is 0.0383 Å. The Os1-C6 bond length (2.180(9) Å) is appreciably longer than both the Os1-C1 bond length (2.040(8) Å) and the Os1-C4 bond length (2.100(7) Å). The three Os-C bond lengths of the metallabicycle all lie in the high end of the reported range for typical Os-C(vinyl)^28^ bond lengths (1.859–2.359 Å) (on the basis of a search of the Cambridge Structural Database, CSD version 5.35, conducted in November, 2013). In addition, the structural parameters indicate a considerable bond distance alternation within the two fused five-membered rings. Therefore, the complex **3a-PF_6_** is not the desired aza-osmapentalene, although the osmabicyclic framework is consistent with the suppositional aza-osmapentalene formula.

The hydroxyl group in **3a-PF_6_** was easily removed to afford the desired complex **4a-(PF_6_)_2_** by treatment of **3a-PF_6_** with NaPF_6_. The complex **4a-(PF_6_)_2_** was isolated as a red solid in 98% yield and was characterised by multinuclear NMR spectroscopy and HRMS. Single-crystal X-ray diffraction established the solid-state structure of **4a-(PF_6_)_2_** unambiguously, demonstrating its aza-osmapentalene arrangement. As shown in Fig. 4c, the two fused five-membered rings of **4a-(PF_6_)_2_** are nearly coplanar. The mean deviation from the least-squares plane through Os1, N1 and C1-C6 is only 0.0154 Å. All the bond distances within the two fused rings fall within the range observed for other typical metallaaromatics^12^,^13^,^14^. The three Os-C distances are nearly equal (Os1-C1 2.041(4), Os1-C4 2.069(4), Os1-C6 2.082(4) Å), and the ring C-C/C-N distances are consistent with extensive delocalisation within the metallacycle (C1-C2 1.407(5), C2-C3 1.410(5), C3-C4 1.394(5), C4-C5 1.381(5), C5-N1 1.374(5), C6-N1 1.396(5) Å). The aromaticity in the metallacyclic rings is also clearly evident in the NMR spectra. In particular, the ^1^H NMR spectrum shows three strongly downfield ^1^H signals corresponding to the ring protons at *δ* = 14.3 (C1*H*), 10.0 (C3*H*), and 9.9 (C5*H*) ppm. With the aid of the ^1^H-^13^C HSQC and ^13^C-DEPT 135 spectra, we observed the signals for the six carbon atoms of the osmabicycle at *δ* = 248.5 (C1), 151.0 (C2), 171.9 (C3), 187.6 (C4), 163.0 (C5) and 234.1 (C6) ppm in the ^13^C{^1^H} NMR spectrum.

Thus, using the strategy outlined in Fig. 2b, we successfully synthesised the first aza-metallapentalene complex in three steps starting from the six-membered osmabenzene complex **1-I**. Further investigations demonstrated that this synthetic strategy could be extended to prepare other aza-osmapentalene complexes with other substituents on the metallacycle. The vinyl-containing aza-osmapentalene **4b-(PF_6_)_2_** was obtained from the reaction of **2-PF_6_** with propynol (Fig. 4a) and was isolated as a brown solid. Similarly, the butenynyl-containing aza-osmapentalene **4c-(PF_6_)_2_** was obtained from the reaction of **2-PF_6_** with penta-1,4-diyn-3-ol and was isolated as a dark-brown solid (Fig. 4a).

**Figure 5:**
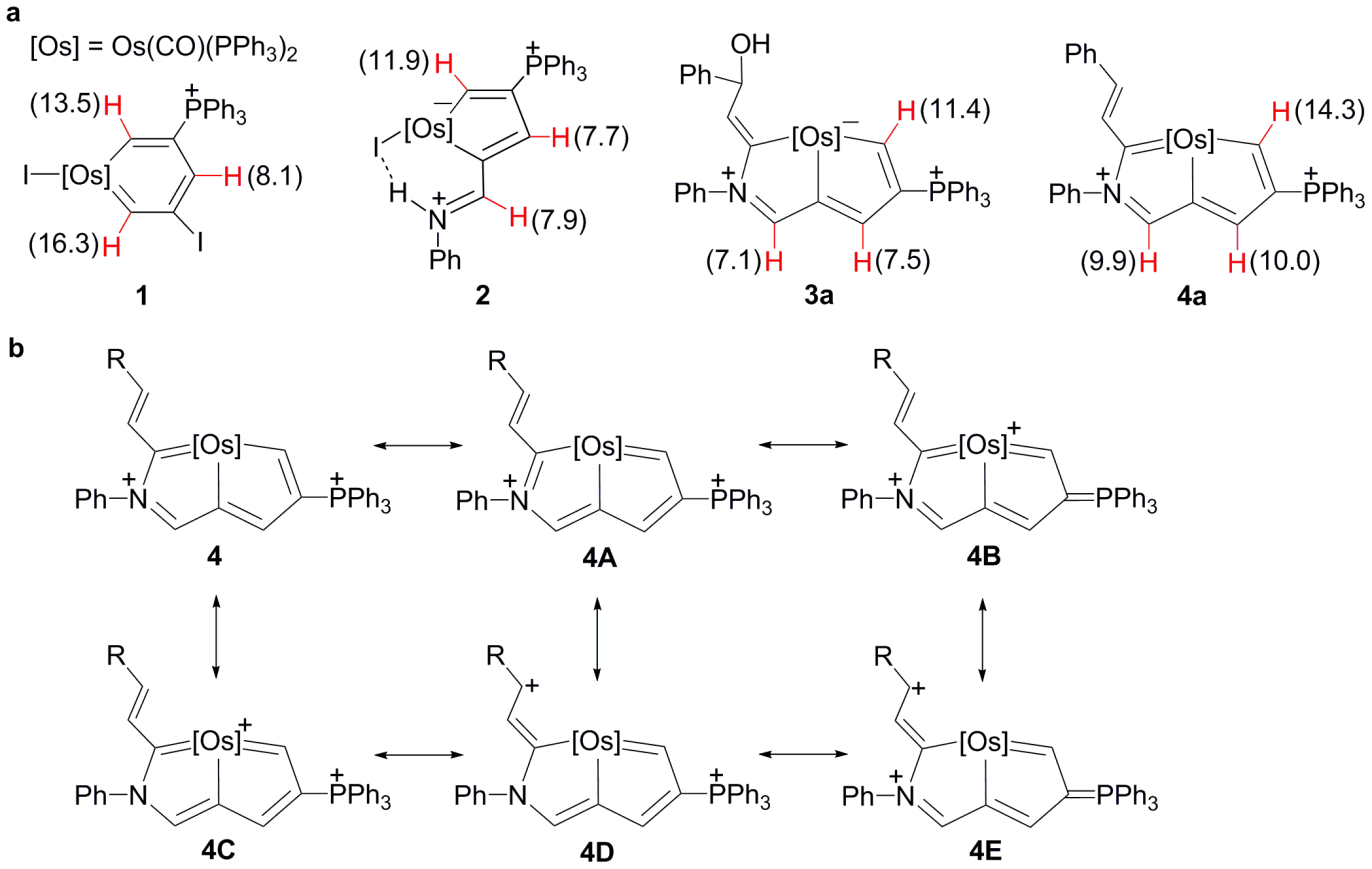
Aromaticity of aza-osmapentalenes: downfield ^1^H chemical shifts and resonance structures. (a) The proton chemical shifts (ppm vs. tetramethylsilane) of the ring protons of osmabenzene **1**, osmapentafulvene **2**, osmabicycle **3a** and aza-osmapentalyne **4a**. (b) Six possible resonance structures for the aza-osmapentalenes **4**.

As previously mentioned, X-ray crystallography revealed a planar structure with no alternation in the carbon–carbon bond lengths of the osmabicyclic rings in the aza-osmapentalene **4a-(PF_6_)_2_**. Protons on the periphery of aromatic compounds are well known to have relatively large downfield NMR spectroscopic chemical shifts due to a diamagnetic ring current. Fig. 5a shows a comparison of the chemical shifts of three specific protons among four different metallacyclic complexes. Compared with the structurally similar complex **3a**, complex **4a** gave considerable downfield chemical shifts because of its aromatic character. The ^1^H NMR chemical shifts as well as the X-ray diffraction data indicate that the metallacycle in the cation of **4-(PF_6_)_2_** has a delocalised structure, with contributions from the six resonance structures **4** to **4E**, as shown in Fig. 5b. Consistent with their aromaticity, all of the new aza-osmapentalenes exhibit remarkably high thermal stability. Solid samples of **4-(PF_6_)_2_** were heated in air at 160°C for at least 5 h without noticeable decomposition. **4-(PF_6_)_2_** was stable even at 80°C in 1,2-dichloro-ethane solution for 5 h. These new aza-osmapentalenes each contain a phosphonium substituent, which is also believed to partially contribute to the high stability^29^.

### DFT calculations of osmabenzene, osmacyclopentadiene and aza-osmapentalene

**Figure 6:**
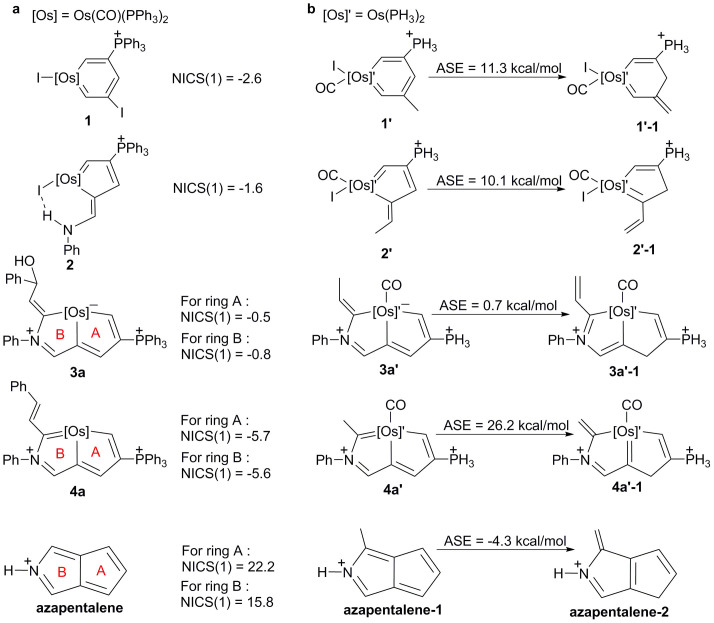
Evaluation of aromaticity for osmabenzene 1, osmapentafulvene 2, osmacycle 3, and aza-osmapentalyne 4a by DFT calculations. (a) The NICS values calculated for the rings in osmabenzene 1, osmapentafulvene 2, osmacycle 3, and aza-osmapentalyne 4a. (b) The ASE values calculated for the model complexes osmabenzene 1′, osmapentafulvene 2′, osmacycle 3′, and aza-osmapentalyne 4a′. The energies computed at the B3LYP level using the LanL2DZ basis set for osmium and the 6-311++G(d,p) basis sets for carbon and hydrogen include zero-point energy corrections.

To gain more insight into the electronic structure and aromaticity of the aza-osmapentalenes **4**, we performed density functional theory (DFT, B3LYP/6-311++G(d,p)) calculations. The nucleus-independent chemical shift (NICS)^30^,^31^,^32^ values were computed for the metallacycle rings of complexes **1-I**, **2-PF6**, and **4-(PF_6_)_2_**. In general, negative NICS values indicate aromaticity, whereas positive values suggest anti-aromaticity. As shown in Fig. 6a, the calculated NICS(1) values are −2.6 and −1.6 ppm for the rings in osmabenzene **1** and osmapentafulvene **2**, respectively. (When the environments at points 1 Å above and below the ring centres are not equivalent, the averaged values were used for NICS(1) values.) These values are comparable to those reported for other metallaaromatics^19^,^20^,^23^,^31^,^33^,^34^,^35^,^36^. The NICS(1) values for the two five-membered rings of aza-osmapentalene **4a** are also negative (−6.7 and −6.9 ppm), in sharp contrast with those of azapentalene (+22.2 and +15.8 ppm). We also evaluated the aromatic stabilisation energy (ASE) by employing the isomerisation method introduced by Schleyer and Pühlhofer^37^. For model complexes **1′**, **2′** and **4a′**, ASE values of 11.3, 10.1 and 26.2 kcal/mol were calculated, respectively; these values contrast sharply with the negative ASE value (−4.3 kcal mol^−1^) calculated for azapentalene. The calculated ASE value for the aza-osmapentalene **4a′** is close to the previously reported values for osmapentalenes^19^,^20^. The negative NICS values and the significant ASE values calculated for these model complexes indicate that aromaticity is closely associated with the metallacycle rings in complexes **1**, **2** and **4a**.

**Figure 7:**
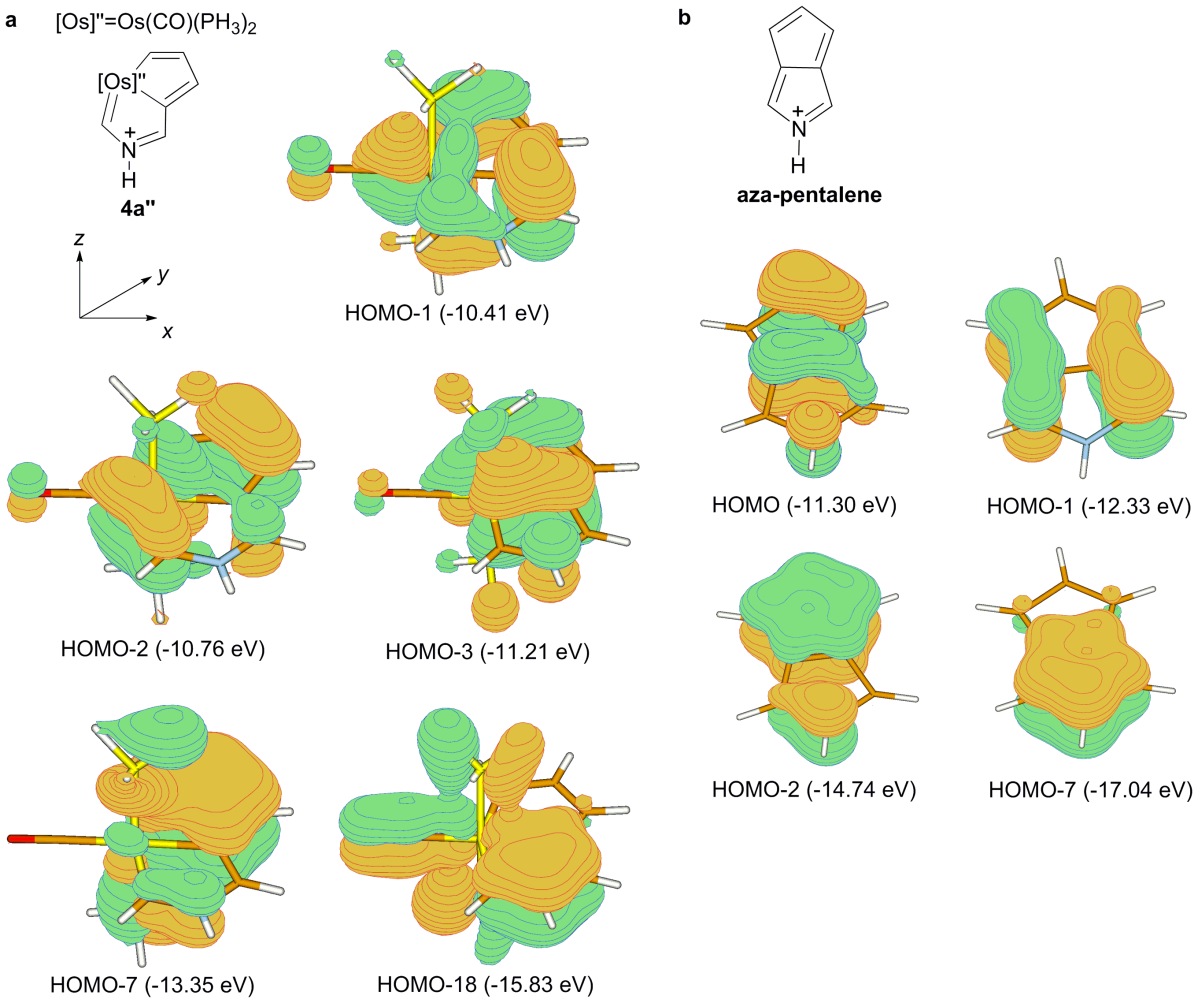
Molecular orbitals calculated for the aza-osmapentalene model 4a″ and azapentalene. The eigenvalues of the MO's are given in parentheses.

Additional evidence for the aromatic nature of aza-osmapentalene **4** was provided by examination of the molecular orbitals calculated for the further simplified model complex **4a″**. π Electrons are normally responsible for aromatic behaviour. Therefore, our attention is paid to those occupied orbitals. As shown in Fig. 7a, the five occupied π molecular orbitals (MOs) calculated for **4a″** (HOMO-1, HOMO-2, HOMO-3, HOMO-7 and HOMO-18) reflect the π-delocalisation along the perimeter of the bicyclic system. HOMO-1, HOMO-3 and HOMO-18 are derived from the orbital interactions between the *p*_π_ atomic orbitals of the C_6_NH_6_ unit (perpendicular to the bicycle plane) and the Os 5*d*_xz_ orbital, whereas HOMO-2 and HOMO-7 are formed by the orbital interactions between the *p*_π_ atomic orbitals of the C_6_NH_6_ unit and the Os 5*d*_yz_ orbital. The approximately uniform contribution of p_π_ orbitals from ring atoms in each of these occupied π molecular orbitals is typical for and consistent with a delocalised π molecular system. The four occupied π MOs calculated for the anti-aromatic azapentalene are also presented in Fig. 7b for comparison.

## Conclusion

We synthesised the first examples of aza-metallapentalenes using metallabenzenes as the starting material. The synthetic route to these aza-metallapentalenes is particularly interesting and remarkable. The bicyclic eight-membered ring complexes are derived from sequential ring contractions, followed by annulation reactions of the six-membered metallabenzenes via the five-membered metallapentafulvenes. Both experimental and theoretical studies suggest that the metallabenzenes, metallapentafulvenes and aza-metallapentalenes all exhibit aromatic character. The present results not only expand the aromaticity concept but also open a promising avenue for the construction of new bicyclic metallaaromatics containing main-group heteroatoms.

## Methods

### General considerations

Unless otherwise stated, all manipulations were carried out at room temperature under a nitrogen atmosphere using standard Schlenk techniques unless otherwise stated. Solvents were distilled from sodium/benzophenone (diethyl ether) or calcium hydride (dichloromethane) under N_2_ prior to use. The starting material [OsCl_2_(PPh_3_)_3_]^38^, HC ≡ CCH(OH)C ≡ CH^39^ and the osmabenzene [Os{CHC(PPh_3_)CHCICH}I_2_(PPh_3_)_2_] was synthesised according to the previously published procedure^24^. Other reagents were used as received from commercial sources without further purification. Further experimental details and the synthetic procedures for **1-I**, **3b-PF_6_**, **3c-PF_6_**, **4b-(PF_6_)_2_** and **4c-(PF_6_)_2_** are described in the Supplementary Methods. The procedures for **2-PF_6_**, **3a-PF_6_**and **4a-(PF_6_)_2_** are described below.

### Synthesis of osmapentafulvene complex 2-PF_6_

Aniline (60 μL, 0.66 mmol) was added to a mixture of **1-I** (300 mg, 0.21 mmol) and sodium hexafluorophosphate (71 mg, 0.42 mmol) in CH_2_Cl_2_ (25 mL). The mixture was heated at reflux for approximately 10 h to afford a purple suspension. The solid suspension was removed by filtration, and the volume of the filtrate was reduced to approximately 2 mL under vacuum. The addition of ether (20 mL) to the solution then produced a purple solid that was collected by filtration, washed with diethyl ether (3 × 2 mL), and dried under vacuum. Yield: 282 mg, 95%. ^1^H NMR (500 MHz, CD_2_Cl_2_): *δ* = 11.9 (d, *J*(PH) = 15.6, 1 H, C^1^*H*), 10.8 (d, *J*(HH) = 14.6, 1 H, N*H*), 7.9 (d, *J*(HH) = 14.6, 1 H, C^5^*H*), 7.7 (br, 1 H, C^3^*H*), 6.6–7.8 ppm (m, 50H, Ph); ^31^P{^1^H} NMR (202 MHz, CD_2_Cl_2_): *δ* = 13.0 (s, C*P*Ph_3_), −3.1 (s, Os*P*Ph_3_), −144.4 ppm (septet, *P*F_6_); ^13^C{^1^H} NMR (126 MHz, CD_2_Cl_2_, plus ^1^H-^13^C HSQC and ^13^C-dept 135): *δ* = 225.2 (br, C^1^), 189.7 (br, C^4^), 175.5 (d, *J*(PC) = 22.0, C^3^), 169.3 (s, C^5^), 158.8 (br, Os(*C*O)), 137.2–118.1 (m, Ph), 122.0 (d, *J*(P,C) = 82.2 Hz, C^2^); HRMS (ESI): [M-PF_6_]^+^ calcd for [C_66_H_54_NP_3_IOOs]^+^, 1288.2072; found, 1288.2091; analysis (calcd., found for C_66_H_54_F_6_INOOsP_4_): C (55.35, 55.24), H (3.80, 3.72), N (0.98, 1.29).

### Synthesis of osmabicyclic complex 3a-PF_6_

A mixture of (Ph)CH(OH)C ≡ CH (40 mg, 0.30 mmol) Ag_2_O (60 mg, 0.26 mmol), NEt_3_ (1 mL) and **2-PF_6_** (365 mg, 0.25 mmol) in dichloromethane (30 mL) was heated at reflux for 3 h to afford a blue suspension (Warning: the reaction may proceed through the formation of silver acetylide, which decomposes violently on contact with moisture and water producing highly flammable and explosive acetylene gas, and causing fire and explosion hazard). The solvent was removed under vacuum, and the residue was extracted with CH_2_Cl_2_ (3 × 5 mL). The volume of the filtrate was filtered through a Celite pad to remove the silver salt and was subsequently reduced to approximately 2 mL under vacuum. Diethyl ether (20 mL) was added slowly with stirring to afford a blue solid, which was collected by filtration, washed with hexane (3 × 3 mL), and dried under vacuum. Yield: 296 mg, 81%. ^1^H NMR (500 MHz, CD_2_Cl_2_): *δ* = 11.4 (d, *J*(P,H) = 20.6 Hz, 1 H, C^1^*H*), 7.5 (br, 1 H, C^3^*H*), 7.1 (s, 1 H, C^5^*H*), 6.9–7.9 (m, 55 H, Ph), 5.9 (d, *J*(H,H) = 8.8 Hz, 1 H, C^7^*H*), 5.3 (d, *J*(H,H) = 8.8 Hz, 1 H, C^8^*H*), 0.9 ppm (br, 1 H, O*H*); ^31^P{1H} NMR (202 MHz, CD_2_Cl_2_): *δ* = 12.2 (s, C*P*Ph_3_), 3.5 (d, *J*(P,P) = 218.6 Hz, Os*P*Ph_3_), 1.0 (*J*(P,P) = 218.6 Hz, Os*P*Ph_3_), −144.4 ppm (septet, *P*F_6_); ^1^C{^1^H} NMR (126 MHz, CD_2_Cl_2_, plus ^13^C-DEPT 135, ^1^H-^13^C HSQC and ^1^H-^13^C HMBC): *δ* = 233.0 (br, C^1^), 196.0 (br, C^4^), 178.6 (br, C^9^), 169.0 (s, C^5^), 163.7 (br, C^6^), 161.5 (d, *J*(P,C) = 25.1 Hz, C^3^), 131.8 (s, C^7^), 123.7 (d, *J*(P,C) = 90.9 Hz, C^2^), 120.6–144.6 (m, Ph), 77.9 (s, C^8^); HRMS (ESI): [M-PF_6_]^+^ calcd for [C_75_H_61_NP_3_O_2_Os]^+^, 1292.3524; found, 1292.3542; analysis (calcd., found for C_75_H_61_F_6_NO_2_OsP_4_): C (62.71, 62.37), H (4.28, 4.19), N (0.98, 1.38).

### Synthesis of aza-osmapentalene complex 4a-(PF_6_)_2_

A mixture of **3a-PF_6_** (200 mg, 0.14 mmol) and sodium hexafluorophosphate (28 mg, 0.17 mmol) in CH_2_Cl_2_ (10 mL) was stirred at room temperature for 30 min to afford a red suspension. The solid suspension was removed by filtration, and the volume of the filtrate was reduced to approximately 2 mL under vacuum. The addition of diethyl ether (20 mL) to the solution then produced a red solid that was collected by filtration, washed with diethyl ether (3 × 2 mL), and dried under vacuum. Yield: 213 mg, 98%. ^1^H NMR (300 MHz, CD_2_Cl_2_): *δ* = 14.3 (d, *J*(P,H) = 16.5 Hz, 1 H, C^1^*H*), 10.0 (br, 1 H, C^3^*H*), 9.9 (s, 1 H, C^5^*H*), 6.4–7.9 (m, 55 H, Ph), 7.7 (d, *J*(H,H) = 15.2, 1 H, C^8^*H*), 6.3 (d, *J*(H,H) = 15.2, 1 H, C^7^*H*); ^31^P{^1^H} NMR (122 MHz, CD_2_Cl_2_): *δ* = 14.7 (s, C*P*Ph_3_), 1.6 ppm (s, Os*P*Ph_3_), −144.4 ppm (septet, *P*F_6_); ^13^C{^1^H} NMR (76 MHz, CD_2_Cl_2_, and ^13^C-DEPT 135, ^1^H-^13^C HSQC and ^1^H-^13^C HMBC): *δ* = 248.5 (br, C^1^), 234.1 (br, C^6^), 191.3 (br, C^9^), 187.6 (br, C^4^), 171.9 (d, *J*(P,C) = 21.4 Hz, C^3^), 163.0 (s, C^5^), 158.9(s, C^8^), 151.0 (d, *J*(P,C) = 67.3 Hz, C^2^), 135.4 (s, C^7^), 117.9–140.5 (m, Ph); HRMS (ESI): [(M-2PF_6_)/2]^+^ calcd for [(C_75_H_60_NP_3_OOs)/2]^+^, 637.6746; found, 637.6763; analysis (calcd., found for C_75_H_60_F_12_NOOsP_5_): C (57.58, 57.86), H (3.87, 3.91), N (0.90, 1.30).

### X-ray Crystallographic Analysis

Single crystals suitable for X-ray diffraction were grown from a dichloromethane solution layered with hexane. Diffraction data were collected on an Oxford Gemini S Ultra charge-coupled device (CCD) area detector (**2-PF_6_, 3a-PF_6_, 4a-(PF_6_)_2_ and 4b-(PF_6_)_2_**) or on a Bruker Apex CCD area detector (**1-I**) using graphite-monochromated Mo Kα radiation (λ = 0.71073 Å) or Cu Kα radiation (λ = 1.54178 Å). Semi-empirical or multi-scan absorption corrections (SADABS) were applied^40^. All structures were solved by the Patterson function, completed by subsequent difference Fourier map calculations, and refined by full-matrix least-squares on *F*^2^ with all the data using the SHELXTL program package^41^. All non-hydrogen atoms were refined anisotropically unless otherwise stated. Hydrogen atoms were placed at idealised positions and were refined using a riding model. See Supplementary Methods for detailed crystal data related to complexes **1-I**, **2-PF_6_**, **3a-PF_6_**, **4a-(PF_6_)_2_** and **4b-(PF_6_)_2_**.

### Computational details

All structures were optimised at the B3LYP level of DFT^42^,^43^,^44^. In addition, the frequency calculations were performed to confirm the characteristics of the calculated structures as minima. In the B3LYP calculations, the effective core potentials (ECPs) of Hay and Wadt with a double-ζ valence basis set (LanL2DZ) were used to describe the Os, P, and I atoms, whereas the standard 6-311++G(d,p) basis set was used for the C and H atoms^45^ for all the ASE calculations. Polarisation functions were added for Os (ζ(f) = 0.886), I (ζ(d) = 0.266) and P (ζ(d) = 0.34)^46^ in all of the calculations. NICS values were calculated at the B3-LYP-GIAO/6-311++G(d,p) level. All the optimisations were performed with the Gaussian 03 software package^47^. See Supplementary Materials for the Cartesian coordinates.

## Author Contributions

H.X. and H.Z. designed the research. T.W., H.H. and J.L. performed the experiments. T.W. and F.H. recorded all NMR data and solved all X-ray structures. H.X., H.Z. and T.W. analysed the experimental data. H.Z. performed the theoretical computations and analysed the theoretical data with assistance from Z.L. and J.Z. H.Z. wrote the paper with assistance from Z.L. All authors discussed the results and contributed to the preparation of the final manuscript.

## Additional information

**Accession codes:** The X-ray crystal structure information is available at the Cambridge Crystallographic Data Centre (CCDC) under deposition numbers CCDC-1013726 (**1-I**), CCDC-1013727 (**2-PF_6_**), CCDC-1013728 (**3a-PF_6_**), CCDC-1013729 (**4a-(PF_6_)_2_**) and CCDC-1013730 (**4b-(PF_6_)_2_**). These data can be obtained free of charge from the Cambridge Crystallographic Data Centre via www.ccdc.cam.ac.uk/data_request/cif.

## Supplementary Material

Supplementary Information Supplementary Information

## Figures and Tables

**Figure 1:**
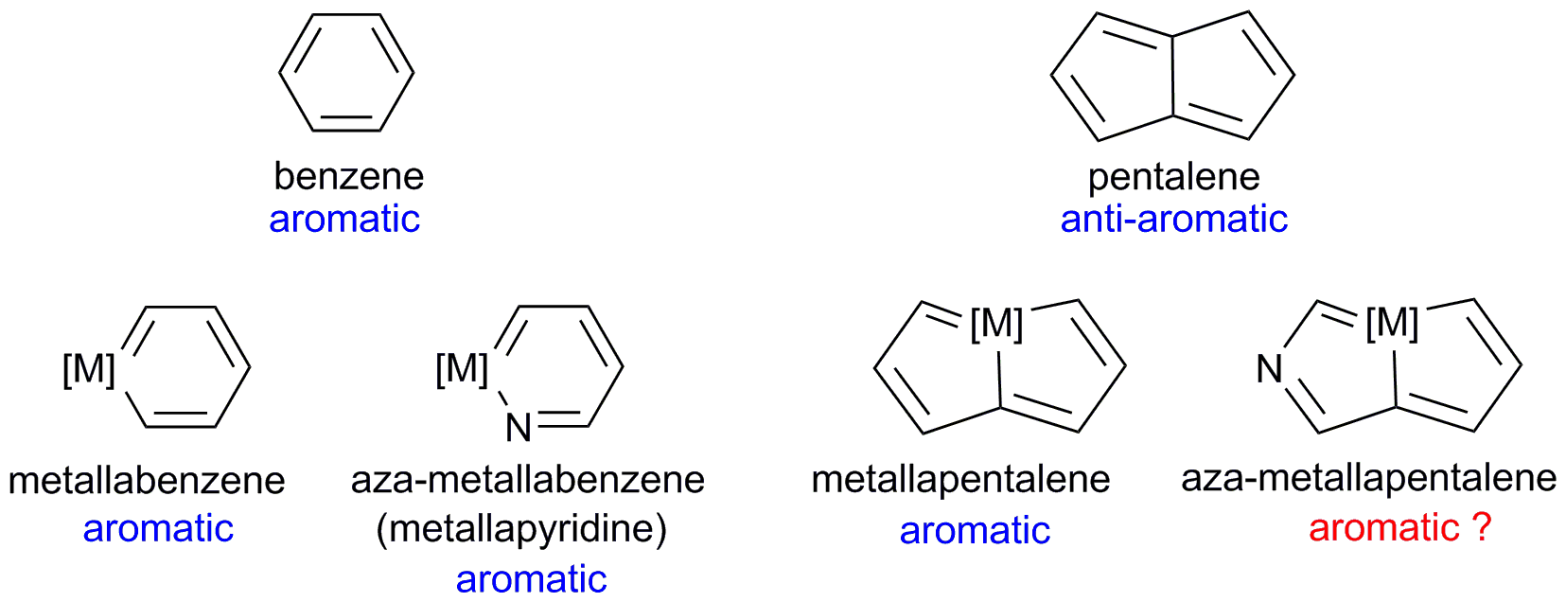
The proposed structure of aza-metallapentalene.
