# Supplementary material for: Synthesis of Aromatic Aza-metallapentalenes from Metallabenzene via Sequential Ring Contraction/Annulation
Source: Sci Rep. 2015 Apr 9;5:9584. doi: 10.1038/srep09584 (PMC4391318; doi:10.1038/srep09584)
Supplement: Supplementary Information [file srep09584-s1.pdf]

Synthesis of Aromatic Aza-metallapentalenes from Metallabenzene via Sequential Ring Contraction/Annulation

Tongdao Wang, Feifei Han, Haiping Huang, Jinhua Li, Hong Zhang,* Jun Zhu, Zhenyang Lin,* and Haiping Xia*

1. Experimental Procedures

All manipulations were carried out at room temperature under a nitrogen atmosphere using standard Schlenk techniques, unless otherwise stated. Solvents were distilled under nitrogen from sodium benzophenone (diethyl ether) or calcium hydride (dichloromethane). Column chromatography was performed on neutral alumina gel (200-300 mesh). The starting material $[Os\{CHC(PPh_3)CHClCH\}I_2(PPh_3)_2]$ was synthesized using the literature procedures.[1] NMR experiments were performed on a Bruker AV-500 spectrometer (^{1}H 500.2 MHz; ^{13}C 125.8 MHz; ^{31}P 202.5 MHz) or a Bruker AV-300 spectrometer (^{1}H 300.1 MHz; ^{13}C 75.5 MHz; ^{31}P 121.5 MHz). ^{1}H and ^{13}C NMR chemical shifts are relative to TMS, and ^{31}P NMR chemical shifts are relative to 85% H_3PO_4. Two-dimensional and one-dimensional NMR are abbreviated as heteronuclear single quantum coherence (HSQC), heteronuclear multiple bond correlation (HMBC), and distortionless enhancement by polarization transfer (DEPT). The absolute values of the coupling constants are given in Hertz (Hz). Multiplicities are abbreviated as singlet (s), doublet (d), triplet (t), multiplet (m) and broad (br). High resolution mass spectra (HRMS) experiments were recorded on a Bruker En Apex Ultra 7.0T FT-MS. Elemental analyses were performed on a Vario EL III elemental analyzer.

Scheme S1. Synthesis of stable osmabenzene **1-I**

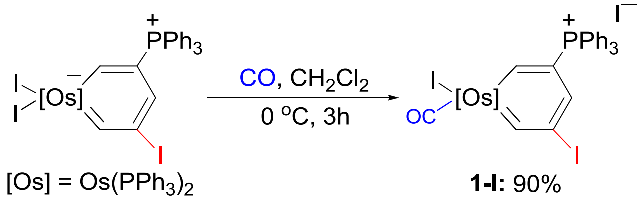

A Solution of $[Os\{CHC(PPh_3)CHCICH\}I_2(PPh_3)_2]$ (660 mg, 0.46 mmol)) in CH_2Cl_2 (50 mL) was stirred at 0 °C under a carbon monoxide atmosphere for approximately 3 h to give a green solution. The solvent was reduced to approximately 5 mL under vacuum, addition of diethyl ether (50 mL) to the solution then produced a green solid that was collected by filtration, washed with diethyl ether (3 × 5 mL), and dried under vacuum. Yield: 606 mg, 90%. ^{1}H NMR (300.1 MHz, CD_2Cl_2): δ = 16.2 (s, 1 H, C^5H), 13.5 (d, J(PH) = 28.3 Hz, 1 H, C^1H), 8.1 (d, J(PH) = 10.0 Hz, 1 H, C^3H), 7.0-7.9 (m, 45 H, ph); $^{31}P\{^1H\}$ NMR (121.5 MHz, CD_2Cl_2): δ = 18.5 (s, CPPh$_3$), -15.1 (s, OsPPh$_3$); $^{13}C\{^1H\}$ NMR (75.5 MHz, CD_2Cl_2): δ = 255.0 (br, C^5), 225.0 (br, C^1), 189.3 (br, Os(CO)), 151.3 (d, J(PC) = 21.9 Hz, C^3), 135.9-118.7 (m, ph), 119.7 (d, J(PC) = 71.5 Hz, C^2), 99.5 (d, J(PC) = 12.8 Hz, C^4); HRMS (ESI): $[M-I]^+$ calcd for $[C_{60}H_{48}P_3I_2OOs]^+$, 1323.0617; found, 1323.0622; analysis (calcd., found for $C_{60}H_{48}I_3OOsP_3$): C (49.74, 49.93), H (3.34, 3.38).

Scheme S2. Synthesis of osmacyclopentadiene **2-PF$_6$**

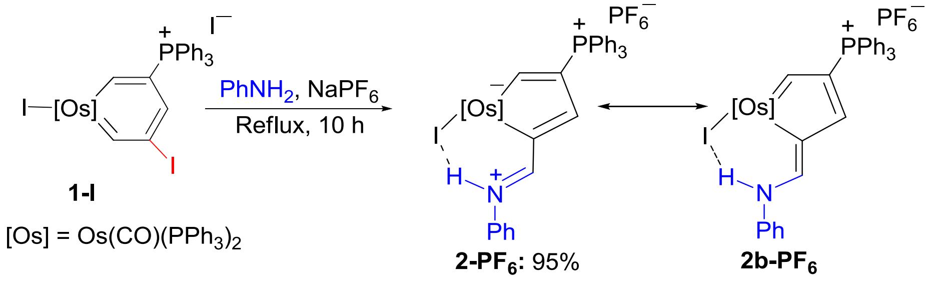

Aniline (60 ul, 0.66 mmol) was added to a mixture of **1-I** (300 mg, 0.21 mmol) and sodium hexafluorophosphate (71 mg, 0.42 mmol) in CH_2Cl_2 (25 mL). The mixture was heated at reflux for approximately 10 h to give a purple suspension. The solid suspension was removed by filtration, and the volume of the filtrate was reduced to approximately 2 mL under vacuum. The addition of ether (20 mL) to the solution then produced a purple solid that was collected by filtration, washed with diethyl ether (3 × 2 mL), and dried under vacuum. Yield: 282 mg, 95%. ^1H NMR (500.2 MHz, CD_2Cl_2): δ = 11.9 (d, J(PH) = 15.6, 1 H, C^1H), 10.8 (d, J(HH) = 14.6, 1 H, N*H*), 7.9 (d, J(PH) = 14.6, 1 H, C^5H), 7.7 (br, 1 H, C^3H), 6.6-7.7 ppm (m, 50H, Ph); $^{31}P\{^1H\}$ NMR (202.5 MHz, CD_2Cl_2): δ = 13.0 (s, C*P*Ph_3), -3.0 (s, Os*P*Ph_3), -144.4 ppm (septet, *P*F_6); $^{13}C\{^1H\}$ NMR (125.8 MHz, CD_2Cl_2, plus ^1H-^{13}C HSQC and ^{13}C-DEPT 135): δ = 225.2 (br, C^1), 189.7 (br, C^4), 175.5 (d, J(PC) = 22.0, C^3), 169.2 (s, C^5), 158.8 (br, Os(*C*O)), 137.2-118.1 (m, Ph), 122.0 (d, J(P,C) = 82.2 Hz, C^2); HRMS (ESI): $[M-PF_6]^+$ calcd for $[C_{66}H_{54}NP_3IOOs]^+$, 1288.2072; found, 1288.2091; analysis (calcd., found for $C_{66}H_{54}F_6INOOsP_4$): C (55.35, 55.24), H (3.80, 3.72), N (0.98, 1.29).

Scheme S3. Synthesis of five-membered osmabicycles **3-PF$_6$**

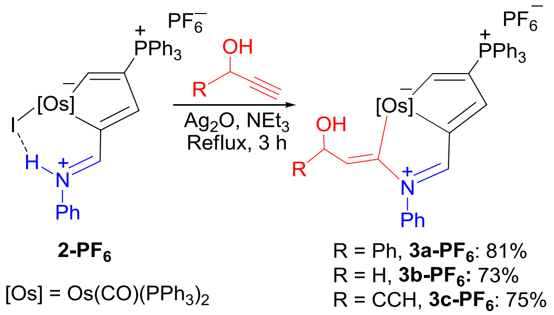

Approximately 1.2 equivalent of terminal alkynol derivatives in CH_2Cl_2 (2 mL) was added to a mixture of **2-PF$_6$** (365 mg, 0.25 mmol) and Ag_2O (60 mg, 0.26 mmol) in CH_2Cl_2 (30 mL). Then

NEt_3(1 mL) was added, and the mixture was heated at reflux for 3 h to afford a blue suspension. The solvent was removed under vacuum and the residue was extracted with CH_2Cl_2 (3 × 5 mL). The volume of the filtrate was filtered through a Celite pad to remove the silver salt and was subsequently reduced to about 2 mL under vacuum. Diethyl ether (20 mL) was added slowly with stirring to afford a blue solid which was collected by filtration, washed with hexane (3 × 3 mL), and dried under vacuum.

3a-PF$_6$, R = Ph, Yield: 296 mg, 81%. ^{1}H NMR (500.2 MHz, CD_2Cl_2): δ = 11.4 (d, *J*(P,H) = 20.6 Hz, 1 H, C^1*H*), 7.5 (br, 1 H, C^3*H*), 7.1 (s, 1 H, C^5*H*), 6.9-7.8 (m, 55 H, Ph), 5.9 (d, *J*(H,H) = 8.8 Hz, 1 H, C^7*H*), 5.3 (d, *J*(H,H) = 8.8 Hz, 1 H, C^8*H*), 0.9 ppm (br, 1 H, O*H*); ^{31}P{^{1}H} NMR (202.5 MHz, CD_2Cl_2): δ = 12.2 (s, C*P*Ph_3), 3.5 (d, *J*(P,P) = 218.6 Hz, Os*P*Ph_3), 1.0 (*J*(P,P) = 218.5 Hz, Os*P*Ph_3), -144.4 ppm (septet, *P*F_6); ^{13}C{^{1}H} NMR (125.8 MHz, CD_2Cl_2, plus ^{13}C-DEPT 135, ^{1}H-^{13}C HSQC and ^{1}H-^{13}C HMBC): δ = 233.0 (br, C^1), 196.0 (br, C^4), 178.6 (br, C^9), 169.0 (s, C^5), 163.8 (br, C^6), 161.5 (d, *J*(P,C) = 25.1 Hz, C^3), 131.8 (s, C^7), 123.7 (d, *J*(P,C) = 90.9 Hz, C^2), 120.7-144.2 (m, Ph), 77.9 (s, C^8); HRMS (ESI): $[M-PF_6]^+$ calcd for $[C_{75}H_{61}NP_3O_2Os]^+$, 1292.3524; found, 1292.3542; analysis (calcd., found for $C_{75}H_{61}F_6NO_2OsP_4$): C (62.71, 62.37), H (4.28, 4.19), N (0.98, 1.38).

3b-PF$_6$, R = H, Yield: 253 mg, 73%. ^{1}H NMR (300.1 MHz, CD_2Cl_2): δ = 11.4 (d, *J*(P,H) = 20.9 Hz, 1 H, C^1*H*), 7.1 (br, 1 H, C^3*H*), 7.0 (s, 1 H, C^5*H*), 6.4-7.7 (m, 50 H, Ph), 5.3 (t, *J*(H,H) = 6.8 Hz, 1 H, C^7*H*), 3.4 (d, *J*(H,H) = 6.8 Hz, 2 H, C^8*H*), 0.8 ppm (br, 1 H, O*H*); ^{31}P{^{1}H} NMR (121.5 MHz, CD_2Cl_2): δ = 12.6 (s, C*P*Ph_3), 2.9 (s, Os*P*Ph_3), -144.5 ppm (septet, *P*F_6); ^{13}C{^{1}H} NMR (75.5 MHz, CD_2Cl_2, plus ^{13}C-DEPT 135, ^{1}H-^{13}C HSQC and ^{1}H-^{13}C HMBC): δ = 231.8 (br, C^1), 195.0 (br, C^4), 179.8 (br, C^9), 169.5 (s, C^5), 163.2 (br, C^6), 161.2 (d, *J*(P,C) = 25.6 Hz, C^3), 129.6 (s, C^7), 125.5 (d, *J*(P,C) = 71.3 Hz, C^2), 120.5-141.0 (m, Ph), 67.5 (s, C^8); HRMS (ESI):

$[M\text{-}PF_6]^+$ calcd for $[C_{69}H_{57}NP_3O_2Os]^+$, 1216.3212; found, 1216.3213; analysis (calcd., found for $C_{69}H_{57}F_6NO_2OsP_4$): C (60.92, 61.01), H (4.22, 4.25), N (1.03, 1.39).

3c-PF$_6$, R = CCH, Yield: 264 mg, 75%. ^{1}H NMR (300.1 MHz, CD_2Cl_2): δ = 11.2 (d, J(P,H) = 20.3 Hz, 1 H, C^1H), 7.5 (br, 1 H, C^3H), 7.0 (s, 1 H, C^5H), 6.8-7.7 (m, 50 H, Ph), 5.3 (d, J(H,H) = 9.7 Hz, 1 H, C^7H), 4.9 (d, J(H,H) = 9.7 Hz, 1 H, C^8H), 2.2 (s, 1 H, C^{10}H), 0.7 ppm (br, 1 H, OH); ^{31}P{^{1}H} NMR (121.5 MHz, CD_2Cl_2): δ = 11.9 (s, CPPh$_3$), 0.88 ppm (s, OsPPh$_3$), -144.5 ppm (septet, PF$_6$); ^{13}C{^{1}H} NMR (75.5 MHz, CD_2Cl_2, plus ^{13}C-DEPT 135, ^{1}H-^{13}C HSQC and ^{1}H-^{13}C HMBC): δ = 233.9 (br, C^1), 196.0 (br, C^4), 177.6 (br, C^{11}), 169.7 (s, C^5), 165.1 (br, C^6), 161.8 (d, J(P,C) = 25.4 Hz, C^3), 130.4 (s, C^7), 123.6 (d, J(P,C) = 91.8 Hz, C^2), 120.2-140.7 (m, Ph), 84.9 (s, C^9), 73.1 (s, C^{10}), 67.5 (s, C^8); HRMS (ESI): $[M\text{-}PF_6]^+$ calcd for $[C_{71}H_{57}NP_3O_2Os]^+$, 1240.3211; found, 1240.3213; analysis (calcd., found for $C_{71}H_{57}F_6NO_2OsP_4$): C (61.60, 61.88), H (4.15, 4.00), N (1.01, 1.40).

Scheme S4. Synthesis of aza-osmacyclpentalene **4-(PF$_6$)$_2$**

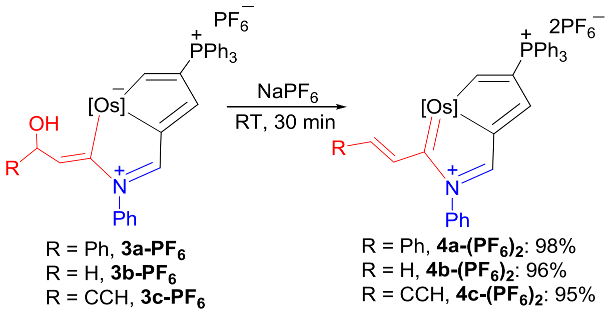

A mixture of **3a-PF$_6$** (200 mg, 0.14 mmol) and sodium hexafluorophosphate (28 mg, 0.17 mmol) in CH_2Cl_2 (10 mL) was stirred at room temperature for 30 min to give a red suspension. The solid suspension was removed by filtration, and the volume of the filtrate was reduced to about 2 mL under vacuum. Addition of diethyl ether (20 mL) to the solution then produced a red solid

that was collected by filtration, washed with diethyl ether (3 × 2 mL), and dried under vacuum. Yield: 213 mg, 98%. ^{1}H NMR (300.1 MHz, CD_2Cl_2): δ = 14.3 (d, *J*(P,H) = 16.5 Hz, 1 H, C^1*H*), 10.0 (br, 1 H, C^3*H*), 9.9 (s, 1 H, C^5*H*), 6.4-7.8 (m, 55 H, Ph), 7.7 (d, *J*(H,H) = 15.2, 1 H, C^8*H*), 6.3 (d, *J*(H,H) = 15.2, 1 H, C^7*H*); ^{31}P{^{1}H} NMR (121.5 MHz, CD_2Cl_2): δ = 14.7 (s, C*P*Ph_3), 1.6 ppm (s, Os*P*Ph_3), -144.4 ppm (septet, *P*F_6); ^{13}C{^{1}H} NMR (75.5 MHz, CD_2Cl_2, plus ^{13}C-DEPT 135, ^{1}H-^{13}C HSQC and ^{1}H-^{13}C HMBC): δ = 248.5 (br, C^1), 234.1 (br, C^6), 191.3 (br, C^9), 187.6 (br, C^4), 171.9 (d, *J*(P,C) = 21.4 Hz, C^3), 163.0 (s, C^5), 158.9(s, C^8), 151.0 (d, *J*(P,C) = 67.2 Hz, C^2), 135.4 (s, C^7), 118.0-140.4 (m, Ph); HRMS (ESI): $[(M\text{-}2PF_6)/2]^+$ calcd for $[(C_{75}H_{60}NP_3OOs)/2]^+$, 637.6746; found, 637.6763; analysis (calcd., found for $C_{75}H_{60}F_{12}NOOsP_5$): C (57.58, 57.86), H (3.87, 3.91), N (0.90, 1.30).

By the same method as that for the formation of **4a-(PF$_6$)$_2$**, from complex **3b-PF$_6$** (230 mg, 0.17 mmol) and sodium hexafluorophosphate (34 mg, 0.20 mmol) in CH_2Cl_2 (10 mL), complex **4b-(PF$_6$)$_2$** was obtained as a brown solid. Yield: 242 mg, 96%. ^{1}H NMR (300.1 MHz, CD_2Cl_2): δ = 14.5 (d, *J*(P,H) = 16.3 Hz, 1 H, C^1*H*), 10.1 (br, 1 H, C^3*H*), 9.9 (s, 1 H, C^5*H*), 6.4-7.8 (m, 50 H, Ph), 6.4 (dd, *J*(H,H) = 15.5 and 1.7 Hz, 1 H, C^8*H*), 5.7-6.0 (m, 2 H, C^7*H* and C^8*H*); ^{31}P{^{1}H} NMR (121.5 MHz, CD_2Cl_2): δ = 14.6 (s, C*P*Ph_3), 0.8 ppm (s, Os*P*Ph_3), -144.4 ppm (septet, *P*F_6); ^{13}C{^{1}H} NMR (75.5 MHz, CD_2Cl_2, plus ^{13}C-DEPT 135, ^{1}H-^{13}C HSQC and ^{1}H-^{13}C HMBC): δ = 249.8 (br, C^1), 236.7 (br, C^6), 192.6 (br, C^9), 186.6 (br, C^4), 171.6 (d, *J*(P,C) = 21.3 Hz, C^3), 162.2 (s, C^5), 152.8 (d, *J*(P,C) = 67.0 Hz, C^2), 144.5 (s, C^7), 140.8(s, C^8), 117.7-140.6 (m, Ph); HRMS (ESI): $[(M\text{-}2PF_6)/2]^+$ calcd for $[(C_{69}H_{56}NP_3OOs)/2]^+$, 599.6589; found, 599.6602; analysis (calcd., found for $C_{69}H_{56}F_{12}NOOsP_5$): C (55.68, 55.33), H (3.79, 3.73), N (0.94, 1.21).

By the same method as that for the formation of **4a-(PF_6)$_2$**, from complex **3c-PF_6** (190 mg, 0.13 mmol) and sodium hexafluorophosphate (27 mg, 0.16 mmol) in CH_2Cl_2 (10 mL), complex **4c-(PF_6)$_2$** was obtained as a dark-brown solid. Yield: 187 mg, 95%. ^{1}H NMR (300.1 MHz, CD_2Cl_2): δ = 14.6 (d, J(P,H) = 16.2 Hz, 1 H, C^1H), 10.1 (br, 1 H, C^3H), 10.0 (s, 1 H, C^5H), 6.4-7.8 (m, 50 H, Ph), 6.7 (dd, J(H,H) = 15.4 and 2.5 Hz, 1 H, C^8H), 6.0 (d, J(H,H) = 15.4 Hz, 1 H, C^7H), 4.0 (d, J(H,H) = 2.5 Hz, 1 H, $C^{10}H$); ^{31}P{^{1}H} NMR (121.5 MHz, CD_2Cl_2): δ = 14.6 (s, CPPh$_3$), 1.0 ppm (s, OsPPh$_3$), -144.3 ppm (septet, PF_6); ^{13}C{^{1}H} NMR (75.5 MHz, CD_2Cl_2, plus ^{13}C-DEPT 135, ^{1}H-^{13}C HSQC and ^{1}H-^{13}C HMBC): δ = 249.8 (br, C^1), 231.7 (br, C^6), 192.9 (br, C^{11}), 186.3 (br, C^4), 171.8 (d, J(P,C) = 21.1 Hz, C^3), 162.4 (s, C^5), 153.3 (d, J(P,C) = 67.1 Hz, C^2), 148.9 (s, C^7), 131.5(s, C^8), 117.6-140.3 (m, Ph), 92.5 (s, C^{10}), 85.0(s, C^9); HRMS (ESI): $[(M-2PF_6)/2]^+$ calcd for $[(C_{71}H_{56}NP_3OOs)/2]^+$, 611.6589; found, 611.6595; analysis (calcd., found for $C_{71}H_{56}F_{12}NOOsP_5$): C (56.39, 56.18), H (3.73, 3.76), N (0.93, 1.23).

2. X-ray Crystallographic Analysis

Single-crystal X-ray diffraction data were collected on an Oxford Gemini S Ultra CCD Area Detector or a Bruker Apex CCD area detector with graphite-monochromated Mo_{Ka} radiation (λ = 0.71073 Å) or Cu_{Ka} radiation (λ = 1.54178 Å). All of the Data were corrected for absorption effects using the multi-scan technique. The structures were solved by direct methods, expanded by difference Fourier syntheses and refined by full matrix least-squares on F^2 using Bruker SHELXTL (Version 6.10) program package. Non-H atoms were refined anisotropically unless otherwise stated. Hydrogen atoms were introduced at their geometric positions and refined as riding atoms unless otherwise stated. The crystal suitable for X-ray diffraction was grown from

CH_2Cl_2 layered with hexane for **1-I**, **2-PF$_6$**, **3a-PF$_6$**, **4a-(PF$_6$)$_2$** and **4b-(PF$_6$)$_2$**. The CH_2Cl_2 molecule in **3a-PF$_6$** was refined with isotropic thermal parameter using fixed C-Cl distances and Cl-C-Cl angle restrains. The counter anion PF_6^- in **3a-PF$_6$** was disordered and two alternative positions were refined to give occupancies of 73.7% for P4 and 26.3% for P4A. Three phenyl groups in **3a-PF$_6$** was also disordered and refined with isotropic thermal parameter. One of CH_2Cl_2 molecules in **4a-(PF$_6$)$_2$** was disordered and refined with partial occupancy factors without addition of H atoms. One of the counter anion PF_6^- in **4a-(PF$_6$)$_2$** was disordered and two alternative positions were refined to give occupancies of 61.9% for P5 and 38.1% for P6. The CH_2Cl_2 molecule in **4b-(PF$_6$)$_2$** was refined with isotropic thermal parameter using fixed C-Cl distances and Cl-C-Cl angle restrains. One of the counter anion PF_6^- in **4b-(PF$_6$)$_2$** was disordered and two alternative positions were refined to give occupancies of 70.7% for P1S and 29.3% for P2S. CCDC-1013726 (**1-I**), CCDC-1013727 (**2-PF$_6$**), CCDC-1013728 (**3a-PF$_6$**), CCDC-1013729 (**4a-(PF$_6$)$_2$**) and CCDC-1013730 (**4b-(PF$_6$)$_2$**) contain the supplementary crystallographic data for this paper. These data can be obtained free of charge from The Cambridge Crystallographic Data Centre via www.ccdc.cam.ac.uk/data_request/cif.

Table S1. Crystal data and structure refinement for **1-I**, **2-PF$_6$**, **3a-PF$_6$**, **4a-(PF$_6$)$_2$** and **4b-(PF$_6$)$_2$**.

	**1-I** ·1.5CH_2Cl_2	**2-PF$_6$** ·1.5CH_2Cl_2	**3a-PF$_6$** ·2CH_2Cl_2	**4a-(PF$_6$)$_2$** ·2CH_2Cl_2	**4b-(PF$_6$)$_2$** ·3CH_2Cl_2
formula	$C_{61.50}H_{51}P_3Cl_3I_3OOs$	$C_{67.50}H_{57}NP_4Cl_3F_6IOOs$	$C_{77}H_{65}NP_4Cl_4F_6O_2Os$	$C_{77.5}H_{64}NP_5Cl_5F_{12}OOs$	$C_{72}H_{62}NP_5Cl_6F_{12}OOs$
Mr	1576.18	1559.47	1606.18	1775.60	1742.98
temp, K	173	130	153	130	173
radiation (Mo or Cu Kα), Å	0.71073	0.71073	1.54178	0.71073	0.71073
crystal system	Triclinic	Monoclinic	Monoclinic	Monoclinic	Triclinic
space group	P-1	P2(1)/*c*	P2(1)	P2(1)/*c*	P-1
a [Å]	10.626(2)	11.6898(4)	13.2494(5)	22.3041(10)	11.8832(7)

b [Å]	12.144(2)	26.5500(11)	23.3493(7)	17.6598(7)	18.5344(12)
c [Å]	24.226(5)	21.4013(11)	13.5865(5)	19.1476(9)	19.1984(10)
α [°]	96.202(6)	90	90	90	107.755(5)
β [°]	102.552(3)	104.812(4)	115.135	98.294(4)	105.415(5)
γ [°]	95.152(4)	90	90	90	94.567(5)
V [Å^3]	3012.7(11)	6421.5(5)	3805.2(2)	7463.1(6)	3822.2(4)
Z	2	4	2	4	2
ρ_{calcd} [gcm^{-3}]	1.738	1.613	1.402	1.580	1.514
μ [mm^{-1}]	3.905	2.752	5.740	2.071	2.054
F(000)	1518	3084	1616	3552	1740
crystal size [mm^3]	0.18×0.15 ×0.04	0.30×0.20×0.10	0.25×0.15×0.05	0.40×0.25×0.20	0.40×0.30×0.05
θ range [°]	0.87 to 25.00	2.73 to 25.00	3.59 to 60.12	2.59 to 25.00	2.95 to 25.00
reflns collected	15427	30434	17339	34248	27678
independent reflns	10468	11283	9322	13114	13440
observed reflns [$I \geq 2\sigma(I)$]	8831	9176	9116	10877	11314
data/restraints/params	10468/15/667	11283/12/775	9322/1438/1071	13114/210/992	13440/633/1087
GOF on F^2	1.000	1.000	1.000	1.000	1.000
R_1/wR_2 [$I \geq 2\sigma(I)$]]	0.0467/0.1375	0.0501/0.1229	0.0482/0.1346	0.0378/0.0792	0.0578/0.1461
R_1/wR_2 (all data)	0.0561/0.1446	0.0667/0.1329	0.0491/0.1358	0.0514/0.0848	0.0717/0.1548
largest peak/hole [e Å^{-3}]	2.884/-1.182	1.638/-1.006	1.746/-1.033	1.131/-0.730	2.007/-0.988

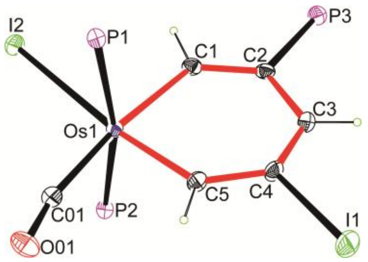

Figure S1. X-ray structure of complex **1-I** drawn with 50% probability. Phenyl moieties in PPh_3, the counter anion and the solvent molecules have been omitted for clarity. Selected bond distances (Å) and angles (deg): Os1-C1 2.065(7), Os1-C5 1.956(7), C1-C2 1.359(10), C2-C3 1.432(11), C3-C4 1.345(10), C4-C5 1.407(11), C4-I1 2.131(7), Os1-C01 1.925(8), C01-O01

1.145(9), Os1-I2 2.8161(8). C5-Os1-C1 86.9(3), C2-C1-Os1 129.6(5), C1-C2-C3 124.4(7), C4-C3-C2 121.4(7), C3-C4-C5 128.4(7), C4-C5-Os1 129.2(6). Estimated standard deviations are given in parenthesis.

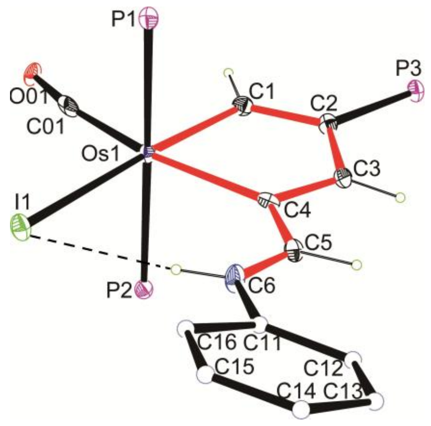

Figure S2. X-ray structure of complex **2-PF$_6$** drawn with 50% probability. Phenyl moieties in PPh_3, the counter anion and the solvent molecules have been omitted for clarity. Selected bond lengths (Å) and angles (deg): Os1-C1 2.004(6), Os1-C4 2.183(7), Os1-I1 2.8347(5), C1-C2 1.372(9), C2-C3 1.430(9), C3-C4 1.386(9), C4-C5 1.418(9), C5-N1 1.309(8), Os1-C01 1.880(9), C01-O01 1.109(8); C1-Os1-C4 77.3(2), Os1-C1-C2 119.9(5), C1-C2-C3 114.1(6), C2-C3-C4 116.9(6), C3-C4-Os1 111.6(5). Estimated standard deviations are given in parenthesis.

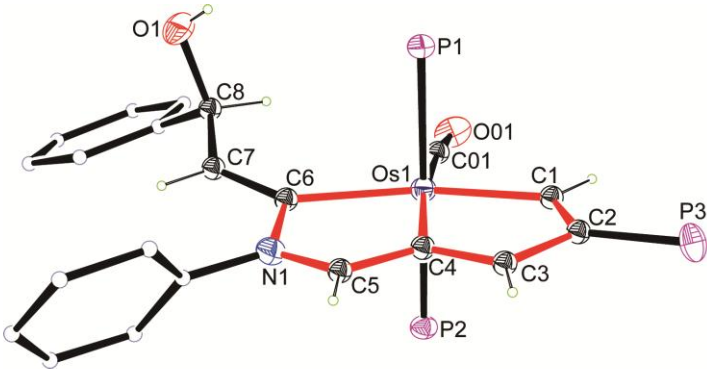

Figure S3. X-ray molecular structure of **3a-PF$_6$** drawn with 50% probability. Phenyl moieties in PPh_3, the counter anion and the solvent molecules have been omitted for clarity. Selected bond lengths (Å) and angles (deg): Os1-C1 2.040(8), Os1-C4 2.100(7), Os1-C6 2.180(9), C1-C2

1.381(11), C2-C3 1.450(11), C3-C4 1.373(12), C4-C5 1.377(13), C5-N1 1.313(11), C6-N1 1.483(11), C6-C7 1.338(12), C7-C8 1.489(12), C8-O1 1.430(12), Os1-C01 1.914(7), C01-O01 1.127(8); C1-Os1-C4 76.1(3), Os1-C1-C2 119.0(6), C1-C2-C3 113.9(7), C2-C3-C4 113.3(7), C3-C4-Os1 117.6(6), C5-C4-Os1 116.5(6), C4-C5-N1 117.9(8), C5-N1-C6 118.4(7), N1-C6-Os1 109.3(5), C6-Os1-C4 77.1(3). Estimated standard deviations are given in parenthesis.

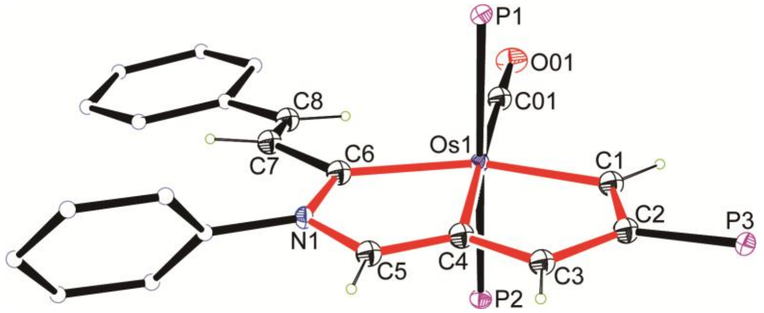

Figure S4. X-ray molecular structure of **4a-(PF_6)$_2$** drawn with 50% probability. Phenyl moieties in PPh_3, the counter anion and the solvent molecules have been omitted for clarity. Selected bond lengths (Å) and angles (deg): Os1-C1 2.041(4), Os1-C4 2.069(4), Os1-C6 2.082(4), C1-C2 1.407(5), C2-C3 1.410(5), C3-C4 1.394(5), C4-C5 1.381(5), C5-N1 1.374(5), C6-N1 1.396(5), C6-C7 1.465(5), C7-C8 1.337(5), C8-C21 1.458(5), Os1-C01 1.920(4), C01-O01 1.153(5); C1-Os1-C4 76.27(15), Os1-C1-C2 117.6(3), C1-C2-C3 114.9(3), C2-C3-C4 112.6(3), C3-C4-Os1 118.5(3), C5-C4-Os1 116.2(3), C4-C5-N1 115.4(3), C5-N1-C6 116.7(3), N1-C6-Os1 114.4(3), C6-Os1-C4 77.31(15). Estimated standard deviations are given in parenthesis.

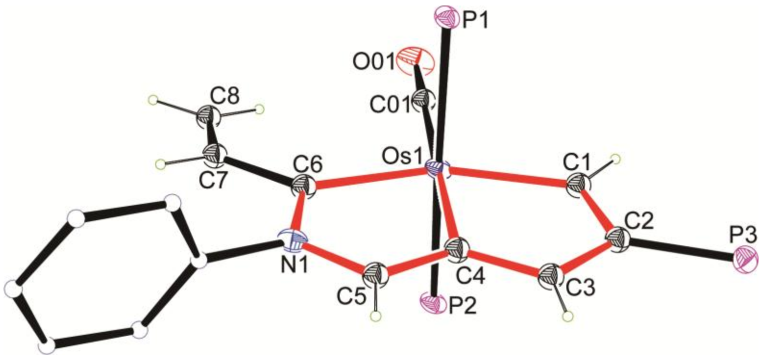

Figure S5. X-ray molecular structure of **4b-(PF_6)$_2$** drawn with 50% probability. Phenyl moieties in PPh_3, the counter anion and the solvent molecules have been omitted for clarity. Selected bond lengths (Å) and angles (deg): Os1-C1 2.042(7), Os1-C4 2.085(7), Os1-C6 2.056(7), C1-C2 1.402(9), C2-C3 1.411(10), C3-C4 1.392(10), C4-C5 1.345(10), C5-N1 1.363(9), C6-N1 1.422(8), C6-C7 1.464(10), C7-C8 1.314(10), Os1-C01 1.917(8), C01-O01 1.161(9); C1-Os1-C4 76.1(3), Os1-C1-C2 118.0(5), C1-C2-C3 115.0(6), C2-C3-C4 112.9(6), C3-C4-Os1 118.0(5), C5-C4-Os1 116.4(5), C4-C5-N1 115.6(6), C5-N1-C6 116.8(6), N1-C6-Os1 113.7(5), C6-Os1-C4 77.3(3). Estimated standard deviations are given in parenthesis.

3. Computational details

All structures were optimised at the B3LYP level of DFT.[2-4] In addition, the frequency calculations were performed to confirm the characteristics of the calculated structures as minima. In the B3LYP calculations, the effective core potentials (ECPs) of Hay and Wadt with a double-ζ valence basis set (LanL2DZ) were used to describe the Os, P, and I atoms, whereas the standard 6-311++G(d,p) basis set was used for the C and H atoms[5] for all the ASE calculations. Polarisation functions were added for Os (ζ(f) = 0.886), I (ζ(d) = 0.266) and P (ζ(d) = 0.34)[6] in all of the calculations. NICS values were calculated at the B3-LYP-GIAO/6-311++G(d,p) level. All the optimisations were performed with the Gaussian 03 software package.[7]

4. Cartesian coordinates together with the electronic energies for all the complexes calculated in this study.

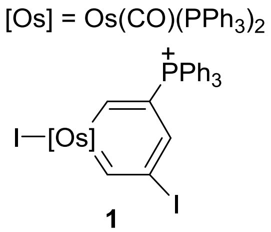

1

E = -2522.823265 a.u.

Os	1.269645	0.065716	0.125266
P	1.205003	2.463332	-0.001036
P	1.732231	-2.280358	-0.082940
P	-3.429185	-0.289007	-0.712551
C	2.964362	0.235070	1.022736
O	3.838146	0.339494	1.753391
I	1.996197	0.181232	-2.593113
I	-1.120302	-0.314467	4.443021
C	-0.669645	-0.140360	-0.558460
H	-0.760610	-0.175833	-1.483452
C	-1.828834	-0.232518	0.141407
C	-1.911919	-0.247346	1.570241
H	-2.746129	-0.307143	1.977065
C	-0.809067	-0.176359	2.339237
C	0.531526	-0.059844	1.931743
H	1.155374	-0.039740	2.621223
C	2.870423	3.249925	0.096519
C	3.053204	4.402749	0.827573
H	2.329305	4.794284	1.260701
C	4.318013	4.990446	0.924183
H	4.440478	5.765454	1.424140
C	5.394511	4.401807	0.262037

H	6.244899	4.773341	0.317510
C	5.174872	3.260917	-0.474184
H	5.883988	2.865717	-0.927429
C	3.944984	2.702392	-0.549333
H	3.827682	1.928301	-1.051543
C	0.308446	3.206505	1.405078
C	0.827318	3.003730	2.691304
H	1.590832	2.485537	2.812093
C	0.201089	3.579203	3.777438
H	0.567541	3.471857	4.624600
C	-0.942304	4.295746	3.636813
H	-1.370548	4.642375	4.385207
C	-1.454421	4.502514	2.410279
H	-2.222325	5.017338	2.313682
C	-0.847594	3.957620	1.290521
H	-1.220702	4.099884	0.450717
C	0.431576	3.273312	-1.445768
C	-0.880339	2.972225	-1.790564
H	-1.312520	2.260990	-1.375401
C	-1.549701	3.719269	-2.746409
H	-2.439108	3.533127	-2.945247
C	-0.901258	4.727326	-3.394095
H	-1.351030	5.233553	-4.031478

C	0.428600	4.999731	-3.105366
H	0.872980	5.675037	-3.567415
C	1.097219	4.279086	-2.140822
H	1.989271	4.463038	-1.953526
C	1.018391	-3.188782	1.351298
C	1.596405	-2.986296	2.579770
H	2.379962	-2.492116	2.650449
C	1.005060	-3.520510	3.716558
H	1.377196	-3.351110	4.552412
C	-0.103983	-4.285771	3.622262
H	-0.486150	-4.647215	4.388329
C	-0.674081	-4.534976	2.376418
H	-1.432836	-5.067060	2.305591
C	-0.094419	-3.974731	1.230791
H	-0.467310	-4.136296	0.393041
C	1.156926	-3.124907	-1.573831
C	-0.174536	-3.092946	-1.953682
H	-0.785376	-2.647058	-1.413662
C	-0.621785	-3.700629	-3.107592
H	-1.523437	-3.669838	-3.330369
C	0.285488	-4.362361	-3.943829
H	-0.007310	-4.769294	-4.726669
C	1.620313	-4.406699	-3.595759

H	2.231593	-4.850489	-4.139473
C	2.048319	-3.781325	-2.418945
H	2.951153	-3.804013	-2.196113
C	3.503475	-2.722998	-0.035911
C	3.907914	-3.942825	0.466307
H	3.275909	-4.524932	0.820824
C	5.229909	-4.309519	0.450936
H	5.486536	-5.146847	0.763845
C	6.180090	-3.423552	-0.035426
H	7.081500	-3.650902	-0.017389
C	5.788288	-2.224115	-0.537086
H	6.426025	-1.641200	-0.885349
C	4.470193	-1.861960	-0.538299
H	4.219841	-1.031998	-0.876510
C	-4.111629	1.347310	-0.863664
C	-4.966531	1.699688	-1.897296
H	-5.188885	1.078085	-2.550938
C	-5.488573	2.991507	-1.951445
H	-6.074688	3.209000	-2.639386
C	-5.173227	3.940997	-1.036440
H	-5.532160	4.797701	-1.095093
C	-4.314164	3.606982	-0.024904
H	-4.066707	4.257869	0.590979

C	-3.815088	2.352638	0.104394
H	-3.272452	2.147035	0.831785
C	-3.318346	-0.932015	-2.365899
C	-3.938979	-2.139607	-2.695081
H	-4.396134	-2.614132	-2.038828
C	-3.882725	-2.631167	-3.961443
H	-4.287075	-3.444788	-4.161933
C	-3.230361	-1.926414	-4.949273
H	-3.205531	-2.253013	-5.819358
C	-2.602321	-0.714806	-4.635706
H	-2.144236	-0.244811	-5.293306
C	-2.662874	-0.225857	-3.369646
H	-2.263027	0.589510	-3.172614
C	-4.539526	-1.318024	0.242337
C	-4.046744	-2.506106	0.798674
H	-3.155608	-2.748522	0.686379
C	-4.910836	-3.308213	1.516692
H	-4.610165	-4.112370	1.873960
C	-6.246658	-2.906563	1.706787
H	-6.818808	-3.440060	2.208778
C	-6.698472	-1.774437	1.179197
H	-7.585864	-1.530718	1.319514
C	-5.883933	-0.953317	0.430067

H	-6.218951	-0.170978	0.054444

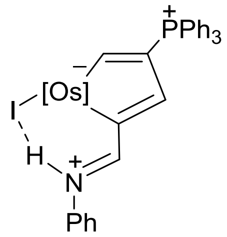

2

E = -2798.355581 a.u.

Os	0.607764	0.094543	-0.908220
P	-3.662217	-0.069480	0.943384
P	0.694796	2.467404	-0.716600
P	0.721135	-2.269614	-0.971029
I	3.441920	0.111186	-0.854218
O	0.023468	0.225663	-3.828445
C	1.749786	3.391931	-1.900000
C	-1.939270	-0.007406	0.556614
C	-3.967039	1.290775	2.063934
C	-0.896706	3.353421	-0.943293
C	-4.588530	0.055289	-0.579593
C	1.894555	4.782230	-1.749294
H	1.478836	5.208225	-1.032782
C	-0.863177	-3.112707	-1.318012
C	1.381049	-2.986988	0.571567
C	-1.383671	-0.007178	-0.698184

H	-1.929276	-0.051297	-1.451156
C	0.355384	0.037934	1.258823
C	-0.983704	0.053555	1.617552
H	-1.246628	0.104697	2.508704
C	3.326688	-0.344435	3.503706
C	-4.392072	1.076582	3.369843
H	-4.587228	0.216861	3.667233
C	-3.215081	-2.276605	2.549121
H	-2.410094	-1.862861	2.772188
C	-5.574215	-3.502425	1.940650
H	-6.387221	-3.906815	1.737952
C	3.280556	4.887068	-3.681694
H	3.810637	5.374453	-4.270220
C	1.733886	-3.063876	-2.271023
C	1.181381	-0.030857	2.411014
H	0.767580	0.007792	3.242103
C	3.160364	-2.987981	-4.207755
H	3.611518	-2.493836	-4.853469
C	-1.824216	2.877624	-1.857065
H	-1.650445	2.083809	-2.309289
C	-4.095279	-1.636384	1.718626
C	4.618085	-0.782889	3.249943
H	4.905538	-0.903818	2.372564

C	0.344295	0.204421	-2.768106
C	-5.292983	-2.267233	1.417464
H	-5.908275	-1.849127	0.860713
C	-2.357977	5.244587	-0.544922
H	-2.539882	6.038172	-0.095995
C	2.398237	2.776997	-2.976407
H	2.333826	1.857160	-3.094849
C	0.626549	-3.047317	1.744078
H	-0.270317	-2.799990	1.730211
C	1.324983	3.048791	0.898539
C	-4.421870	-0.995219	-1.492266
H	-3.906133	-1.734679	-1.265238
C	-1.178824	4.567871	-0.301085
H	-0.562585	4.920930	0.300220
C	-5.027612	-0.925226	-2.740572
H	-4.915894	-1.612752	-3.356458
C	-3.007004	3.560131	-2.120114
H	-3.616649	3.221478	-2.736193
C	1.818985	-4.449017	-2.293450
H	1.361136	-4.950398	-1.656032
C	3.129754	3.533561	-3.855072
H	3.531904	3.120447	-4.586218
C	-3.676349	2.587847	1.640916

H	-3.389903	2.730875	0.767559
C	-3.268817	4.743101	-1.459814
H	-4.058651	5.203970	-1.628220
C	2.641424	5.524627	-2.635786
H	2.716073	6.444349	-2.531182
C	2.697878	3.259561	1.071932
H	3.269135	3.219756	0.339584
C	2.407356	-2.340565	-3.248712
H	2.350850	-1.411974	-3.256654
C	-4.524159	2.173225	4.231074
H	-4.812627	2.041019	5.106260
C	-1.448591	-2.841645	-2.573433
H	-1.056272	-2.217610	-3.139957
C	-5.966388	1.206719	-2.158218
H	-6.475468	1.949144	-2.392984
C	-5.802131	0.183254	-3.057215
H	-6.214247	0.232888	-3.889373
C	-5.373881	1.150197	-0.891495
H	-5.505688	1.832030	-0.274935
C	5.479637	-1.037216	4.308964
H	6.358607	-1.286848	4.139434
C	3.249302	-4.358298	-4.213759
H	3.758309	-4.789804	-4.860539

C	2.879086	-0.197135	4.803840
H	2.016849	0.101675	4.984556
C	-1.510095	-4.029747	-0.493687
H	-1.148028	-4.234612	0.339324
C	0.489248	3.058502	2.017274
H	-0.419674	2.888743	1.917730
C	-4.695352	-4.150455	2.744756
H	-4.890478	-4.997078	3.076442
C	2.581569	-5.093369	-3.258216
H	2.641596	-6.023244	-3.259417
C	3.201234	3.524384	2.331213
H	4.107316	3.705696	2.438354
C	2.514381	-3.808682	2.974431
H	2.891370	-4.095737	3.775535
C	-3.220882	-4.372165	-2.149938
H	-4.003987	-4.790978	-2.419747
C	2.730836	-3.321652	0.651622
H	3.261845	-3.270301	-0.109570
C	1.002999	3.317547	3.282856
H	0.440692	3.352143	4.024538
C	-3.505073	-3.525924	3.059846
H	-2.896012	-3.949755	3.619152
C	1.205146	-3.472153	2.930336

H	0.688839	-3.526731	3.701754
C	-2.691047	-4.642246	-0.898881
H	-3.125521	-5.235145	-0.327860
C	-4.227550	3.451255	3.792363
H	-4.311838	4.172518	4.371359
C	3.289382	-3.730418	1.843910
H	4.191141	-3.953805	1.881061
C	2.368304	3.524610	3.428298
H	2.724332	3.662693	4.275757
C	3.746769	-0.502163	5.815270
H	3.454040	-0.426775	6.694585
C	-3.808913	3.651488	2.490042
H	-3.616073	4.511361	2.193261
C	-2.606801	-3.500386	-2.975954
H	-2.959830	-3.337004	-3.819811
C	5.054356	-0.926702	5.574696
H	5.623494	-1.128016	6.281746
N	2.483096	-0.145584	2.380620
H	2.863382	-0.097382	1.610839

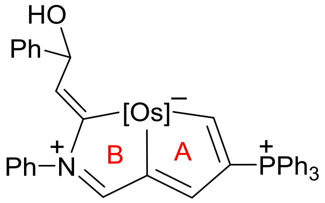

3a

E = -3209.118440 a.u.

Os	-0.466362	0.044572	-0.445667
P	-0.321541	2.406886	-0.222396
P	-0.636073	-2.316444	-0.649804
P	4.147998	-0.183124	0.379711
C	1.556205	-0.004656	-0.705299
H	1.924950	0.074017	-1.555634
C	2.370824	-0.154854	0.400470
C	1.646008	-0.319897	1.644974
H	2.062614	-0.466809	2.463410
C	0.283777	-0.237521	1.495309
C	-0.647489	-0.364547	2.501278
H	-0.385775	-0.517362	3.381024
C	-2.264163	0.108993	0.785952
C	-3.541273	0.489497	0.676857
H	-4.090422	0.377981	1.418103
C	-2.901021	-0.442387	3.232392
C	-3.853384	-1.465729	3.101219
H	-3.878032	-2.013173	2.349996
C	-4.762173	-1.621150	4.144075
H	-5.409059	-2.288684	4.085517
C	-4.731732	-0.816940	5.260773
H	-5.363238	-0.937561	5.932531

C	-3.793162	0.144089	5.390287
H	-3.765361	0.666601	6.159985
C	-2.846999	0.362997	4.348542
H	-2.206758	1.033963	4.420078
C	-5.345039	0.578895	-1.029870
C	-6.380978	0.223498	-0.174478
H	-6.243036	0.216510	0.745818
C	-7.623561	-0.122254	-0.693738
H	-8.317291	-0.361142	-0.120379
C	-7.830204	-0.112607	-2.068389
H	-8.660988	-0.342288	-2.415555
C	-6.792554	0.244664	-2.923038
H	-6.931494	0.252058	-3.842786
C	-5.551774	0.588475	-2.404437
H	-4.858202	0.827513	-2.976424
C	1.169793	3.192913	-0.985115
C	1.905114	4.208325	-0.381847
H	1.703863	4.475407	0.485176
C	2.941793	4.821135	-1.075622
H	3.434194	5.499062	-0.672696
C	3.243078	4.418557	-2.372727
H	3.936861	4.829353	-2.836840
C	2.507391	3.405659	-2.975569

H	2.708676	3.138671	-3.842598
C	1.470785	2.792825	-2.281732
H	0.978344	2.115015	-2.684726
C	-0.347471	2.923810	1.520858
C	0.856868	2.921382	2.255263
H	1.668470	2.718690	1.848061
C	0.786868	3.232065	3.606449
H	1.576875	3.234552	4.097861
C	-0.356077	3.522762	4.241200
H	-0.360890	3.715040	5.151605
C	-1.550906	3.535589	3.491257
H	-2.352610	3.772047	3.897393
C	-1.525147	3.197711	2.161240
H	-2.322477	3.154151	1.686107
C	-1.544097	3.529050	-1.012759
C	-1.853907	4.776331	-0.451230
H	-1.440351	5.037788	0.339855
C	-2.752277	5.610141	-1.048651
H	-2.989481	6.411441	-0.636012
C	-3.309556	5.271541	-2.258549
H	-3.921547	5.845102	-2.662793
C	-2.971056	4.110367	-2.865921
H	-3.327729	3.909671	-3.700623

C	-2.099352	3.212199	-2.260129
H	-1.888319	2.410237	-2.678886
C	0.842875	-3.120888	-1.417671
C	1.456730	-4.256600	-0.903797
H	1.120356	-4.659548	-0.135386
C	2.572113	-4.791433	-1.537984
H	2.982912	-5.551607	-1.193761
C	3.074494	-4.188426	-2.686266
H	3.820871	-4.547805	-3.110310
C	2.460565	-3.052690	-3.200202
H	2.795885	-2.649311	-3.967987
C	1.345182	-2.517858	-2.566015
H	0.934265	-1.757651	-2.908798
C	-0.903369	-3.302418	0.870302
C	0.111658	-3.404680	1.823693
H	0.933432	-2.995258	1.675334
C	-0.110526	-4.112804	2.988674
H	0.572040	-4.188637	3.617365
C	-1.343778	-4.715231	3.233628
H	-1.482244	-5.182247	4.027129
C	-2.347429	-4.624615	2.317651
H	-3.169070	-5.028142	2.481423
C	-2.127475	-3.913251	1.115246

H	-2.806947	-3.853739	0.483267
C	-1.987671	-2.942085	-1.721582
C	-3.160020	-2.243523	-1.864382
H	-3.249078	-1.416823	-1.447256
C	-4.219913	-2.742323	-2.618623
H	-5.009164	-2.259109	-2.705431
C	-4.078085	-3.965705	-3.229828
H	-4.780384	-4.308220	-3.734081
C	-2.928822	-4.679671	-3.109654
H	-2.841144	-5.494850	-3.549073
C	-1.883287	-4.193635	-2.328513
H	-1.115385	-4.704345	-2.210203
C	4.708363	1.507182	1.109880
C	5.688427	2.248733	0.460862
H	6.053479	1.940666	-0.336367
C	6.123009	3.453767	1.004003
H	6.778185	3.950711	0.569760
C	5.575758	3.915403	2.195499
H	5.865883	4.720414	2.558484
C	4.595728	3.173946	2.844512
H	4.230602	3.482037	3.641678
C	4.160972	1.971371	2.300418
H	3.507663	1.473839	2.736215

C	4.676481	-1.828495	1.261143
C	3.937646	-2.968984	0.969915
H	3.219871	-2.922370	0.380525
C	4.272662	-4.183528	1.559681
H	3.778343	-4.946606	1.365214
C	5.344282	-4.256773	2.440447
H	5.568127	-5.069100	2.834871
C	6.083117	-3.116284	2.731676
H	6.800967	-3.162921	3.321127
C	5.749203	-1.902095	2.142702
H	6.243466	-1.138990	2.337247
C	4.767907	-0.221456	-1.287057
C	4.590448	0.868816	-2.117700
H	4.168350	1.628308	-1.786091
C	5.013894	0.858891	-3.394930
H	4.892703	1.611489	-3.927835
C	5.635926	-0.282514	-3.928774
H	5.903144	-0.308601	-4.819401
C	5.833954	-1.324589	-3.121523
H	6.289977	-2.064316	-3.453938
C	5.395491	-1.355002	-1.822834
H	5.511535	-2.116616	-1.303070
N	-1.917196	-0.267970	2.179413

O	-4.494750	2.448520	-0.297251
H	-3.833500	2.857105	-0.040501
C	-0.946006	0.091382	-2.297943
O	-1.119418	0.116156	-3.410762
C	-4.147092	1.083357	-0.547400
H	-3.424825	0.878276	-1.309747

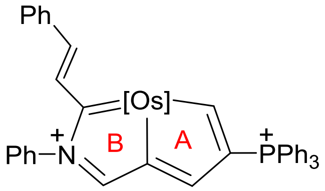

4a

E = -2902.096384 a.u.

Os	0.917172	0.110395	-0.531008
P	1.197842	-2.284099	-0.692501
P	0.630489	2.493504	-0.619452
P	-3.606768	-0.274435	0.703021
C	-1.114807	-0.077770	-0.596392
H	-1.562825	-0.149774	-1.407157
C	-1.813085	-0.095243	0.619138
C	-1.003667	-0.014434	1.772341
H	-1.340427	-0.023924	2.638526
C	0.353659	0.081859	1.475732
C	1.345497	0.177653	2.378288
H	1.183355	0.191354	3.292651

C	2.719062	0.273150	0.445467
C	4.116314	0.351191	0.012835
H	4.783851	0.274699	0.655792
C	4.468627	0.526721	-1.240756
H	3.817874	0.606009	-1.898525
H	5.368957	0.570787	-1.467865
C	3.740984	0.269575	2.783879
C	4.399579	1.443633	3.056229
H	4.129362	2.245499	2.672346
C	5.490465	1.391505	3.927795
H	5.948728	2.173537	4.138213
C	5.900585	0.177727	4.486776
H	6.648356	0.150211	5.040416
C	5.209685	-0.959083	4.225100
H	5.469285	-1.757637	4.622888
C	4.114517	-0.939751	3.364599
H	3.641687	-1.719922	3.181833
C	0.371771	-3.303887	0.579182
C	0.603410	-3.015710	1.950883
H	1.204928	-2.349005	2.192604
C	-0.072063	-3.739927	2.934296
H	0.073246	-3.548499	3.831210
C	-0.961673	-4.745269	2.576826

H	-1.427586	-5.206533	3.236274
C	-1.155660	-5.064911	1.244802
H	-1.723812	-5.763438	1.012784
C	-0.502853	-4.345075	0.255319
H	-0.649773	-4.558272	-0.637646
C	2.958707	-2.771134	-0.706521
C	3.582978	-3.339573	0.392402
H	3.091770	-3.543919	1.155540
C	4.946128	-3.601044	0.346522
H	5.367309	-3.973992	1.086824
C	5.681910	-3.320007	-0.776965
H	6.592777	-3.511958	-0.797671
C	5.067414	-2.747075	-1.878751
H	5.563518	-2.540929	-2.637377
C	3.716733	-2.486850	-1.845863
H	3.303240	-2.116741	-2.591889
C	0.582881	-3.028047	-2.263722
C	-0.421182	-2.433452	-3.004897
H	-0.770875	-1.614199	-2.742938
C	-0.901641	-3.065538	-4.141778
H	-1.565228	-2.658137	-4.648492
C	-0.398246	-4.300097	-4.528179
H	-0.739134	-4.724373	-5.281351

C	0.594717	-4.889489	-3.803158
H	0.935657	-5.713989	-4.069750
C	1.102328	-4.265665	-2.665076
H	1.783143	-4.667806	-2.173154
C	-0.348963	3.168660	0.779155
C	-1.632745	3.608265	0.596617
H	-1.989171	3.641145	-0.260243
C	-2.409822	4.004019	1.679933
H	-3.287750	4.285995	1.550166
C	-1.861883	3.974279	2.953763
H	-2.377972	4.237413	3.681016
C	-0.592051	3.568724	3.151039
H	-0.233191	3.569670	4.009675
C	0.184832	3.148382	2.072565
H	1.056406	2.855996	2.211294
C	-0.284066	3.116183	-2.067342
C	-0.935351	2.284693	-2.986807
H	-0.874319	1.359893	-2.901395
C	-1.662791	2.831440	-4.009935
H	-2.061804	2.271298	-4.637957
C	-1.815231	4.191842	-4.128359
H	-2.356569	4.543385	-4.796175
C	-1.171205	5.024262	-3.264334

H	-1.256436	5.945101	-3.356894
C	-0.390495	4.509363	-2.249449
H	0.067505	5.084945	-1.681297
C	2.185652	3.447657	-0.724098
C	2.702339	4.232364	0.316689
H	2.210662	4.337777	1.099836
C	3.937471	4.854350	0.194472
H	4.278213	5.364607	0.894164
C	4.653002	4.705300	-0.972762
H	5.484163	5.112840	-1.057017
C	4.144343	3.953973	-2.026479
H	4.632234	3.868404	-2.814484
C	2.920427	3.336534	-1.904296
H	2.581662	2.841328	-2.615529
C	-4.113509	-0.748051	2.360979
C	-3.876870	0.154197	3.419114
H	-3.502669	0.989462	3.252678
C	-4.212127	-0.218661	4.720307
H	-4.065869	0.363533	5.430316
C	-4.768063	-1.468721	4.937226
H	-4.980828	-1.724013	5.806178
C	-5.009778	-2.336015	3.906573
H	-5.402839	-3.162552	4.076286

C	-4.674060	-1.986010	2.618471
H	-4.824148	-2.582338	1.920892
C	-4.043382	-1.557607	-0.461399
C	-3.367290	-2.772358	-0.376414
H	-2.697352	-2.877851	0.261145
C	-3.672505	-3.828120	-1.221279
H	-3.231244	-4.641348	-1.140540
C	-4.644023	-3.650612	-2.188183
H	-4.844462	-4.348274	-2.769859
C	-5.325404	-2.446222	-2.305299
H	-5.976011	-2.335663	-2.960487
C	-5.019321	-1.400424	-1.427877
H	-5.475833	-0.592073	-1.494817
C	-4.438812	1.246289	0.224140
C	-5.382543	1.845632	1.051588
H	-5.581629	1.464904	1.875953
C	-6.026988	3.014273	0.652885
H	-6.654666	3.415538	1.208469
C	-5.730573	3.572265	-0.574442
H	-6.170639	4.346766	-0.845136
C	-4.775762	2.991047	-1.412498
H	-4.576098	3.380986	-2.233082
C	-4.128458	1.830937	-1.014553

H	-3.490875	1.440362	-1.567280
C	1.427348	0.146205	-2.378576
O	1.727023	0.166899	-3.501273
N	2.606136	0.252861	1.863694

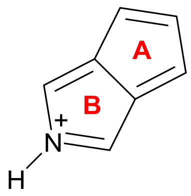

azapentalene

E = -324.763584 a.u.

C	-1.371288	-1.126111	-0.000095
C	0.013839	-0.693193	-0.000125
C	0.062303	0.702959	0.000002
C	2.240037	0.023085	0.000144
C	1.447419	1.135944	0.000097
H	-1.688290	-2.168247	-0.000018
H	3.326813	-0.020736	0.000087
H	1.764353	2.178037	0.000046
C	1.400228	-1.242284	0.000148
H	1.708851	-2.266809	0.000305
C	-1.324098	1.252043	-0.000095
H	-1.632732	2.276565	-0.000150
N	-2.163921	-0.013267	-0.000095
H	-3.142193	-0.220592	-0.000075

[Os]' = $Os(PH_3)_2$

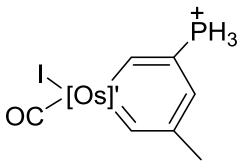

1'

E = -472.699047 a.u.

Os	-0.111140	-0.539668	0.011026
C	-0.992601	-2.260159	0.052401
O	-1.356079	-3.358994	0.058461
I	-2.437528	1.201005	-0.012515
C	1.014919	1.199001	-0.027388
H	0.448267	2.132234	-0.058437
C	2.389897	1.353238	0.000254
C	3.309634	0.253812	0.014414
H	4.373629	0.466419	0.057394
C	2.912629	-1.062322	-0.025353
C	1.577387	-1.535921	-0.032727
H	1.545853	-2.626673	-0.075753
P	-0.340224	-0.477854	2.308808
H	0.616182	-1.242579	2.877124
H	-0.213429	0.795271	2.739592
H	-1.557305	-0.950129	2.652565
P	-0.433169	-0.565403	-2.276273
H	0.498468	-1.354011	-2.853031

H	-1.663765	-1.047142	-2.552063
H	-0.322408	0.689905	-2.760457
C	4.042337	-2.108404	-0.058092
H	4.950395	-1.642755	-0.379810
H	4.178601	-2.516971	0.921400
H	3.782943	-2.892308	-0.738613
P	3.185147	2.990079	-0.026741
H	4.525711	2.840010	0.026788
H	2.849570	3.634708	-1.164433
H	2.770041	3.709660	1.037397

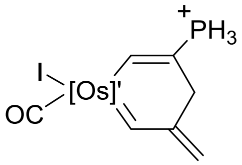

1'-1

E = -472.681018 a.u.

Os	-0.116604	-0.579440	-0.003203
C	-1.314236	-2.092434	0.176642
O	-2.021532	-2.985968	0.282861
I	-2.308171	1.268490	0.026739
C	1.144946	1.101768	-0.260761
H	0.679021	2.014004	-0.641661
C	2.469166	1.153468	0.006045
C	2.860834	-1.362109	0.065583

C	1.455606	-1.707123	-0.087877
H	1.364030	-2.771947	-0.326249
P	-0.153220	-0.158689	2.373914
H	0.745175	-0.861721	3.230090
H	0.096987	1.176130	2.785881
H	-1.379814	-0.414920	3.037388
P	-0.558757	-0.551982	-2.374714
H	0.209971	-1.389102	-3.235797
H	-1.873300	-0.904162	-2.771489
H	-0.405737	0.694500	-3.036426
P	3.320451	2.705541	-0.281821
H	3.926453	3.303330	0.857199
H	4.400479	2.653731	-1.204959
H	2.430067	3.678746	-0.785703
C	3.794161	-2.292519	-0.226143
H	3.509585	-3.292030	-0.535274
H	4.858452	-2.089918	-0.164021
C	3.281923	0.004084	0.580667
H	3.172339	0.009663	1.674134
H	4.352025	0.135008	0.394648

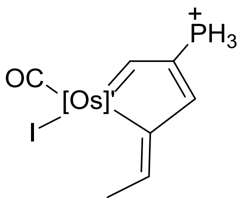

2'

E = -472.685123 a.u.

Os	0.120894	-0.373293	-0.000405
I	-2.494775	0.162587	-0.002953
O	-0.617375	-3.342208	-0.035177
C	2.940987	0.190678	0.006532
C	1.984867	-0.872859	0.000425
H	2.391781	-1.890974	0.000719
C	0.967084	1.526715	0.017798
C	2.423497	1.479117	0.018333
H	3.064359	2.364640	0.029691
C	0.566565	2.847144	0.018200
H	1.361636	3.602444	0.013739
C	-0.378074	-2.219644	-0.016526
P	-0.203481	-0.106198	-2.371392
H	-0.709347	1.151200	-2.784990
H	0.923533	-0.220903	-3.240173
H	-1.108116	-0.992696	-3.011556
P	-0.215746	-0.168340	2.372924
H	0.902877	-0.331510	3.244250
H	-0.700914	1.083724	2.826147

H	-1.141714	-1.056133	2.979111
P	4.705389	-0.105316	-0.006425
H	5.392853	0.253543	-1.196944
H	5.440023	0.552701	1.013933
H	4.971447	-1.483089	0.163841
C	-0.791183	3.429356	0.019716
H	-1.586643	2.691897	-0.037046
H	-0.917926	4.017052	0.943296
H	-0.884567	4.163699	-0.788762

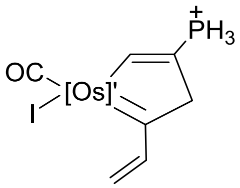

2'-1

E = -472.669073 a.u.

Os	0.077932	-0.446304	0.010099
I	-2.656994	0.299008	-0.007809
O	-0.071227	-3.423375	0.043394
C	2.907067	0.353224	-0.006189
C	2.062094	-0.726827	-0.058340
H	2.408199	-1.588619	-0.122542
C	0.852817	1.593772	0.034886
C	0.334726	2.914643	-0.002961
H	0.937964	3.617547	0.070941

C	-0.122984	-2.316644	0.056255
P	0.004855	-0.405273	-2.298380
H	0.305962	0.835772	-2.736150
H	0.900282	-1.286394	-2.792683
H	-1.234387	-0.741217	-2.715419
P	-0.007529	-0.318757	2.314991
H	0.927782	-1.134790	2.845825
H	0.230368	0.951749	2.704502
H	-1.230683	-0.698691	2.741661
P	4.726286	0.307062	-0.032876
H	5.175958	0.928697	-1.143671
H	5.208453	0.934977	1.060622
H	5.143867	-0.976729	-0.035374
C	-0.978892	3.218131	-0.140331
H	-1.374553	4.109860	0.299183
H	-1.622609	2.563776	-0.690192
C	2.238318	1.613241	0.080663
H	2.578474	2.218098	-0.733796
H	2.499546	2.081925	1.006404

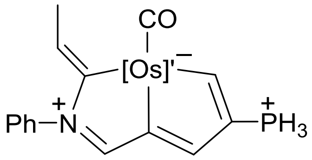

3a'

Os	0.938765	-0.590667	0.031912

C	2.671430	0.485498	0.050727
H	3.494509	0.054162	0.092216
C	2.604939	1.864561	-0.000774
C	1.252699	2.386875	-0.005813
H	1.055933	3.295857	-0.002189
C	0.291678	1.406478	-0.016727
C	-1.069732	1.611242	-0.015137
H	-1.425719	2.470919	-0.009831
C	-1.224309	-0.783585	-0.160502
C	-2.123302	-1.721986	-0.474995
H	-3.021054	-1.482633	-0.460882
C	-3.285301	0.769501	0.026347
C	-4.017075	0.217654	1.090020
H	-3.606300	-0.295257	1.747914
C	-5.386013	0.468806	1.113142
H	-5.900171	0.113436	1.803682
C	-6.002135	1.228729	0.144842
H	-6.921276	1.364522	0.181578
C	-5.284468	1.779529	-0.856576
H	-5.706080	2.312118	-1.492830
C	-3.882645	1.543620	-0.943845
H	-3.381513	1.904276	-1.639557
N	-1.850303	0.554933	-0.016558

C	1.724395	-2.316790	0.289707
O	2.285463	-3.281399	0.443918
P	0.771788	-0.519314	2.334764
H	1.998198	-0.666062	2.879633
H	0.251060	0.667835	2.711620
H	-0.031479	-1.518015	2.758862
P	1.075311	-0.663079	-2.272911
H	1.160876	-1.947870	-2.678487
H	-0.026729	-0.095016	-2.807058
H	2.171586	0.011329	-2.680163
P	4.032382	2.993203	-0.031938
H	3.960864	3.779060	-1.127299
H	4.020939	3.763929	1.076371
H	5.174161	2.273801	-0.068004
C	-1.777651	-3.171641	-0.863099
H	-1.254050	-3.641525	-0.056904
H	-2.679151	-3.711296	-1.065510
H	-1.159593	-3.169329	-1.736540

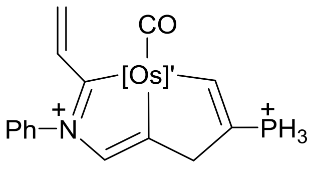

3a'-1

Os	0.931250	-0.616680	0.019510

C	2.659763	0.465733	0.054669
H	3.484447	0.037018	0.091145
C	2.588042	1.845104	0.021794
C	0.276568	1.378457	-0.002716
C	-1.085617	1.577985	0.000265
H	-1.444898	2.436147	0.016859
C	-1.230883	-0.815224	-0.177699
C	-3.297985	0.727312	0.028086
C	-4.028667	0.158308	1.083438
H	-3.616566	-0.361903	1.734737
C	-5.398578	0.403899	1.108570
H	-5.912037	0.037241	1.793708
C	-6.016671	1.174522	0.150032
H	-6.936360	1.306297	0.187671
C	-5.300152	1.741581	-0.843099
H	-5.723187	2.281133	-1.472505
C	-3.897353	1.512227	-0.932132
H	-3.396935	1.884191	-1.622383
N	-1.862136	0.518822	-0.016265
C	1.723234	-2.343129	0.254687
O	2.287843	-3.307593	0.396381
P	0.761780	-0.577189	2.322946
H	1.988217	-0.726630	2.867023

H	0.236147	0.602746	2.715327
H	-0.038066	-1.584605	2.732654
P	1.070295	-0.657321	-2.285943
H	1.161169	-1.936159	-2.708807
H	-0.033396	-0.086277	-2.813462
H	2.164372	0.026724	-2.682904
P	4.011183	2.979503	0.007379
H	3.937713	3.779857	-1.077303
H	3.995719	3.735084	1.126020
H	5.155742	2.265017	-0.037273
C	-1.816589	-3.022478	-0.864274
H	-0.836761	-3.410759	-0.679677
H	-2.554675	-3.638869	-1.333520
C	-2.125973	-1.752698	-0.505795
H	-3.171770	-1.526533	-0.498162
C	1.233818	2.362275	0.022460
H	1.067674	2.972290	-0.840777
H	1.084100	2.946556	0.906259

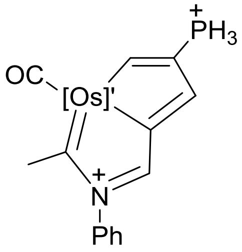

4a'

E = -785.433084 a.u.

Os	-0.886088	-0.612249	0.000038
C	-2.657560	0.418749	0.000069
H	-3.677911	0.028408	0.000190
C	-2.529091	1.828867	-0.000081
C	-1.218405	2.363881	-0.000168
H	-1.004338	3.431228	-0.000214
C	-0.243517	1.363089	-0.000122
C	1.127656	1.547552	-0.000204
H	1.674716	2.485238	-0.000449
C	1.202188	-0.826272	0.000137
C	3.333469	0.567696	-0.000111
C	4.005068	0.658837	-1.216345
H	3.464467	0.587098	-2.153100
C	5.386998	0.843372	-1.209012
H	5.921923	0.917447	-2.147986
C	6.075607	0.935188	-0.000192
H	7.149131	1.079764	-0.000231
C	5.386809	0.845378	1.208676
H	5.921595	0.920991	2.147607
C	4.004882	0.660863	1.216087
H	3.464123	0.590629	2.152867
C	-1.630507	-2.413580	0.000132

O	-2.130730	-3.444428	0.000174
N	1.874364	0.408951	-0.000063
P	-0.886185	-0.711152	2.417874
H	-0.846414	0.518392	3.131059
H	-2.005083	-1.331800	3.032746
H	0.177761	-1.410548	3.047251
P	-3.984899	2.912882	-0.000173
H	-4.073566	3.773045	1.123267
H	-4.074555	3.771617	-1.124631
H	-5.161770	2.132380	0.000810
P	-0.886473	-0.711355	-2.417794
H	0.178071	-1.409660	-3.047375
H	-2.004846	-1.333246	-3.032362
H	-0.848149	0.518171	-3.131088
C	2.139870	-2.035261	0.000636
H	2.756988	-2.008358	-0.873059
H	2.757074	-2.007570	0.874244
H	1.561312	-2.935355	0.001070

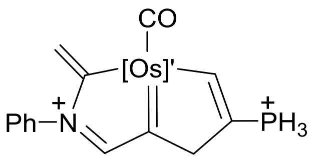

4a'-1

E = -785.391362 a.u.

Os	-0.857349	-0.594675	0.023014
C	-2.696242	0.417137	-0.114334
H	-3.692070	-0.029819	-0.166918
C	-2.602469	1.779234	-0.139389
C	1.167662	1.518827	0.039940
H	1.651980	2.484641	-0.071012
C	1.267580	-0.833309	0.188274
C	3.377248	0.579792	0.032334
C	4.124035	-0.224173	-0.848253
H	3.625619	-0.943277	-1.488714
C	5.501827	-0.041261	-0.929103
H	6.081625	-0.644948	-1.619762
C	6.132501	0.922100	-0.136072
H	7.207679	1.056509	-0.200129
C	5.382235	1.715200	0.742501
H	5.876374	2.447514	1.372805
C	4.003293	1.557687	0.824697
H	3.425709	2.144711	1.532234
C	-1.418151	-2.503912	0.005519
O	-1.713400	-3.614675	-0.005811
N	1.950808	0.435951	0.095098
P	-1.219038	-0.735710	2.402018
H	-1.657469	0.440225	3.068330

H	-2.210834	-1.652878	2.837665
H	-0.135780	-1.111216	3.242197
P	-4.075230	2.787104	-0.267209
H	-4.304895	3.683146	0.810952
H	-4.143000	3.636468	-1.403915
H	-5.222657	1.966314	-0.333080
P	-0.747755	-0.819273	-2.373832
H	-0.875183	-2.129098	-2.904743
H	-1.710813	-0.129520	-3.159882
H	0.444388	-0.402448	-3.027107
C	2.037442	-1.881430	0.591631
H	1.606960	-2.876252	0.643264
H	3.068192	-1.787216	0.925387
C	-1.222856	2.422739	-0.067265
H	-1.001071	3.049284	-0.947785
C	-0.235212	1.284576	0.027750
H	-1.107667	3.097881	0.797872

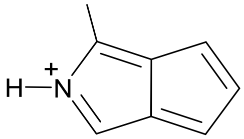

azapentalene-1

E = -364.077791 a.u.

C	1.282435	-0.247698	0.000013
C	-0.078409	-0.450326	-0.000062

C	-0.709008	0.830023	0.000143
C	-2.354075	-0.775595	-0.000124
C	-2.129154	0.611167	0.000042
H	-3.322410	-1.252665	-0.000254
H	-2.900565	1.370823	0.000042
C	-1.117520	-1.442550	-0.000199
H	-0.985590	-2.517172	-0.000281
C	0.281087	1.785309	0.000218
H	0.244267	2.865100	-0.000369
N	1.493050	1.119086	0.000211
H	2.399675	1.565626	0.000268
C	2.470620	-1.227400	-0.000112
H	2.426166	-1.842072	0.874589
H	2.423675	-1.844869	-0.872710
H	3.387577	-0.675960	-0.002302

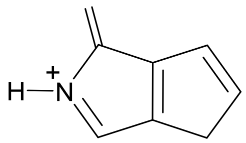

azapentalene-2

E = -364.084622 a.u.

C	1.354342	-0.377975	-0.001534
C	-0.016387	-0.496209	0.007305
C	-0.566769	0.820615	0.005166

C	-2.307745	-0.680376	0.022086
H	-3.287725	-1.081946	-0.292668
C	-1.114723	-1.422386	0.017921
H	-1.049356	-2.503093	0.022131
C	0.480366	1.712951	-0.005051
H	0.510241	2.792943	-0.010510
N	1.648888	0.973189	-0.009008
H	2.581322	1.362907	-0.016358
C	2.344735	-1.303007	-0.003698
H	3.369433	-0.995051	-0.011238
H	2.101167	-2.344900	0.002154
C	-1.997687	0.689844	0.014403
H	-2.404141	1.154090	0.888571
H	-2.419944	1.141988	-0.858618

[Os]"=Os(CO)(PH_3)$_2$

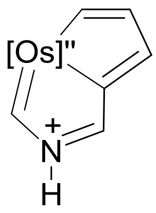

4a"

E = -507.766016 a.u.

Os	0.278660	0.014546	0.020790
C	-0.144113	-0.074390	-1.995155
H	0.543777	-0.077533	-2.845644

C	-1.517478	-0.150782	-2.339999
C	-2.424702	-0.167339	-1.277003
H	-3.501942	-0.224514	-1.435973
C	-1.815145	-0.106220	0.000861
C	-2.415954	-0.111905	1.235543
H	-3.470547	-0.166877	1.493642
C	-0.180797	0.021036	2.002711
C	2.212732	0.156935	0.057252
O	3.364508	0.249398	0.090464
N	-1.508636	-0.043342	2.278095
P	0.470961	-2.377374	-0.066545
H	-0.619779	-3.086878	-0.639576
H	1.543081	-2.917761	-0.825071
H	0.631881	-3.118102	1.136613
P	0.082383	2.403001	-0.157540
H	-0.205108	3.168961	1.005719
H	1.199538	3.128276	-0.650459
H	-0.936544	2.889124	-1.021137
H	-1.859295	-0.196206	-3.372727
H	0.407159	0.061616	2.923287
H	-1.853437	-0.045819	3.236948

5. NMR spectra

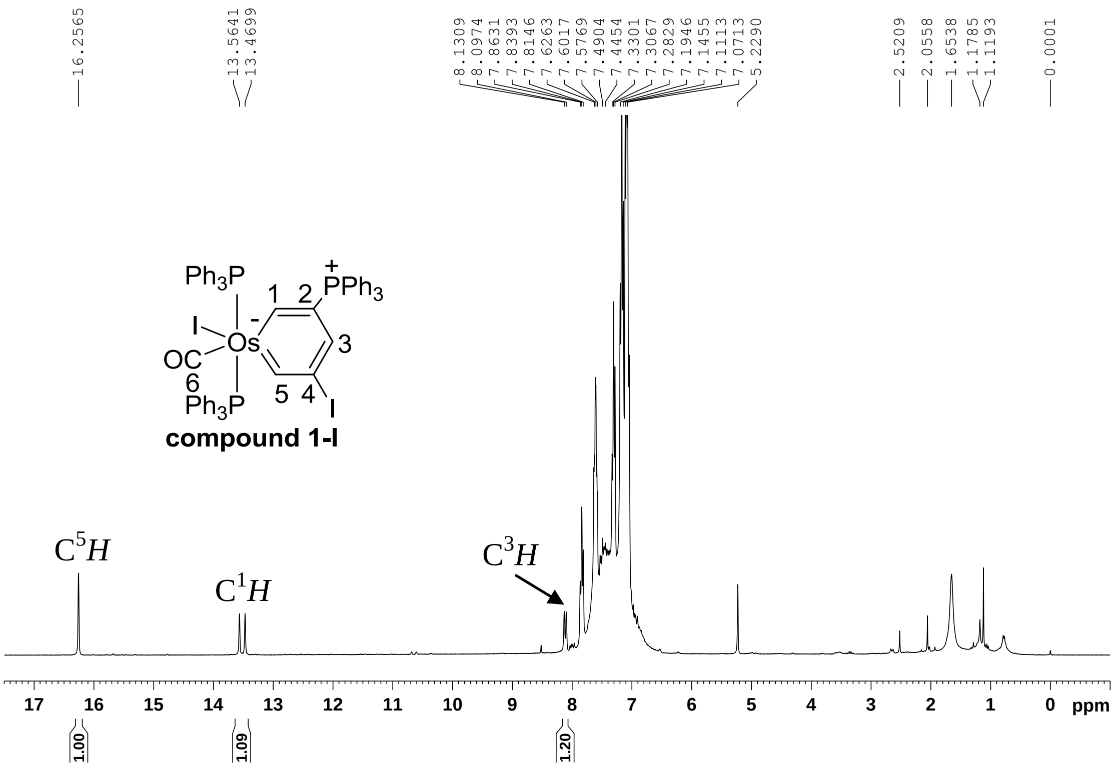

Figure S6. The ^{1}H NMR (300.1 MHz, CD_2Cl_2) Spectrum for compound **1-I** at room temperature.

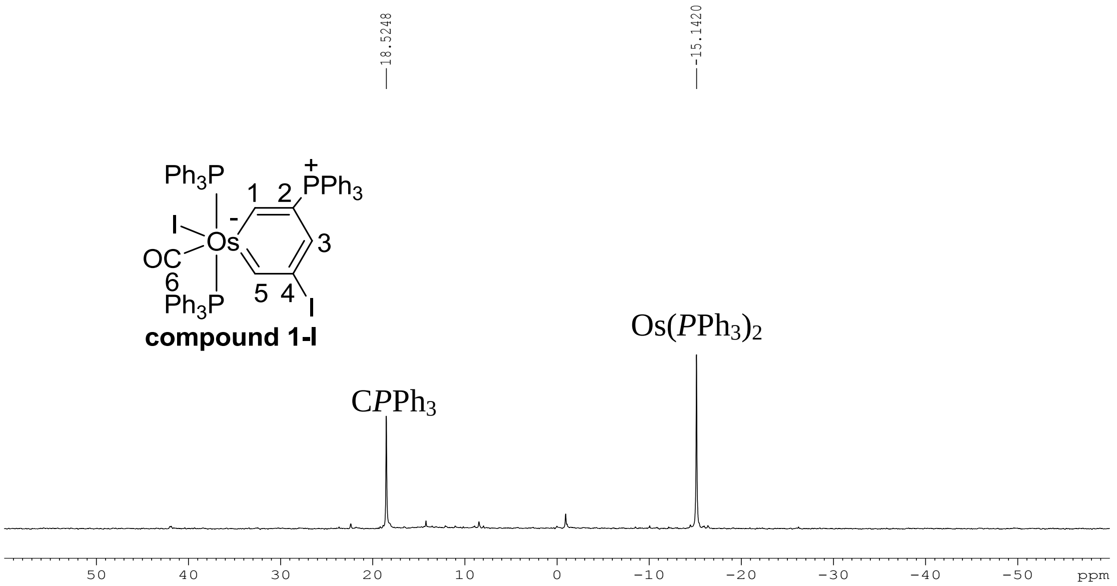

Figure S7. The $^{31}P\{^{1}H\}$ NMR (121.5 MHz, CD_2Cl_2) Spectrum for compound **1-I** at room temperature.

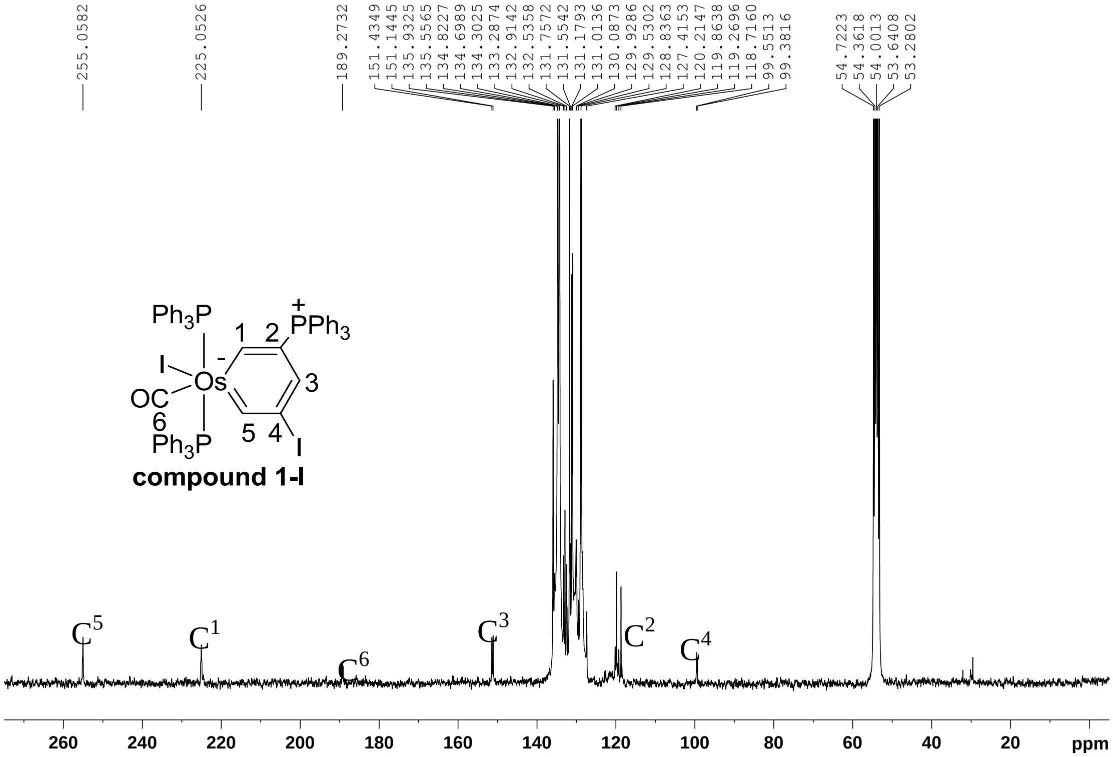

Figure S8. The $^{13}C\{^1H\}$ NMR (75.5 MHz, CD_2Cl_2) Spectrum for compound **1-I** at room temperature.

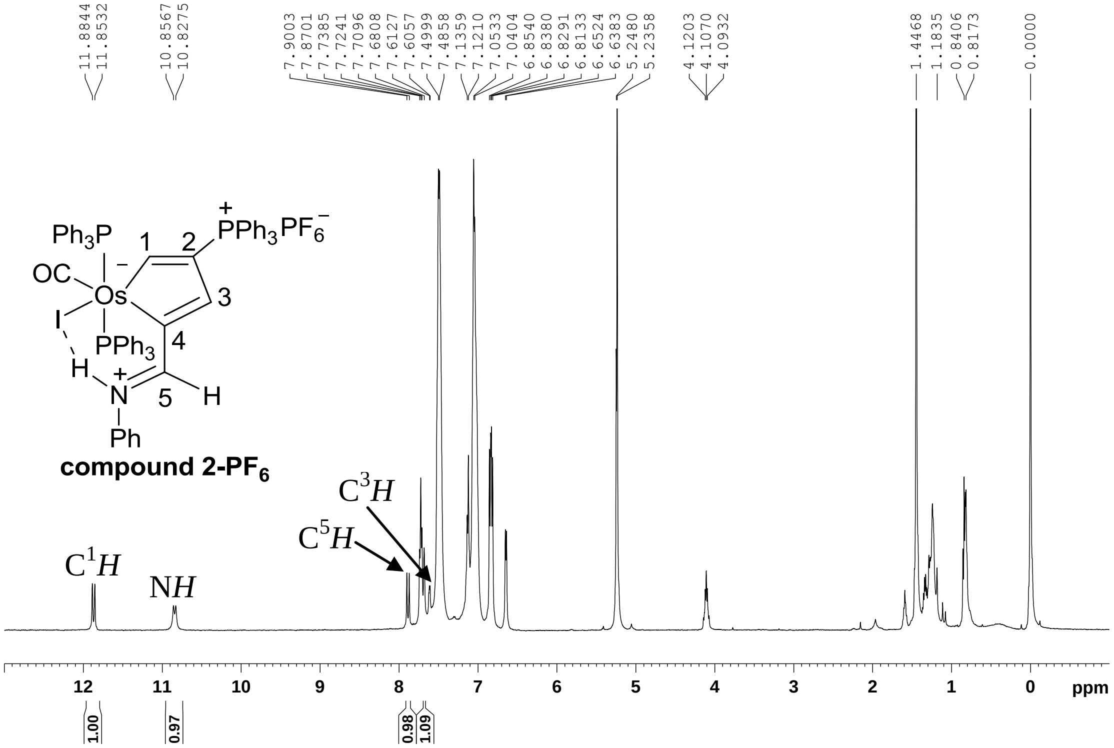

Figure S9. The ^1H NMR (500.2 MHz, CD_2Cl_2) Spectrum for compound **2-PF$_6$** at room temperature.

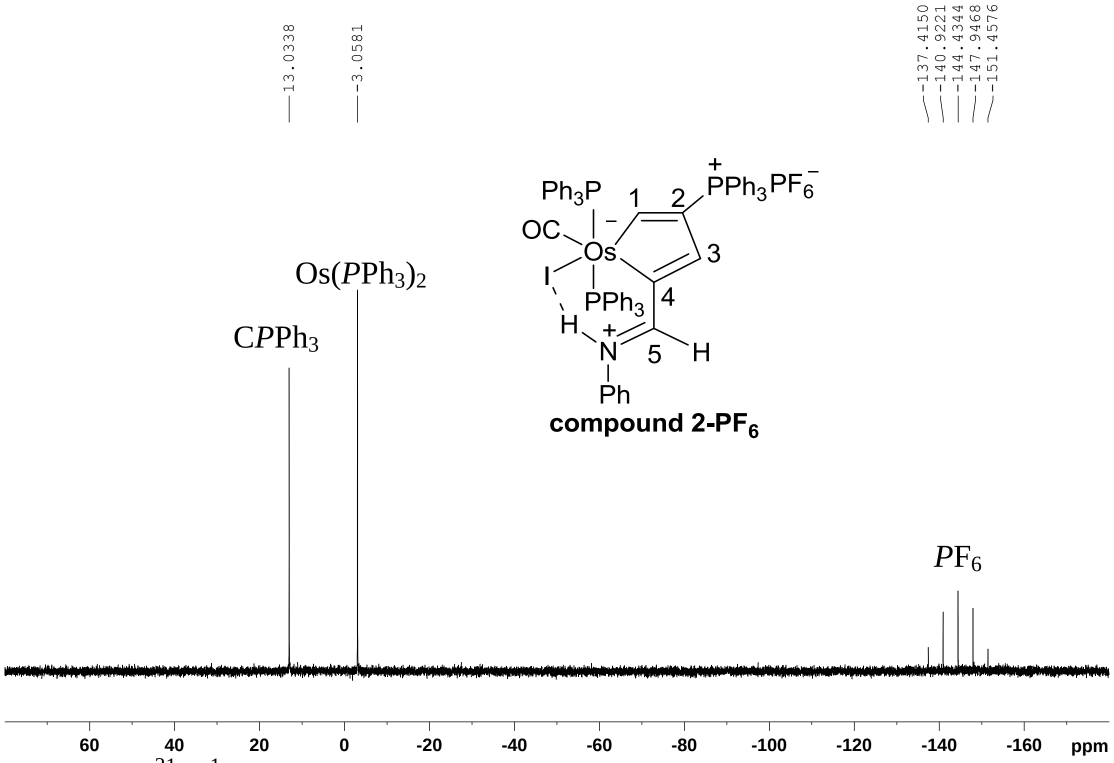

Figure S10. The $^{31}P\{^{1}H\}$ NMR (202.5 MHz, CD_2Cl_2) Spectrum for compound **2-PF$_6$** at room temperature.

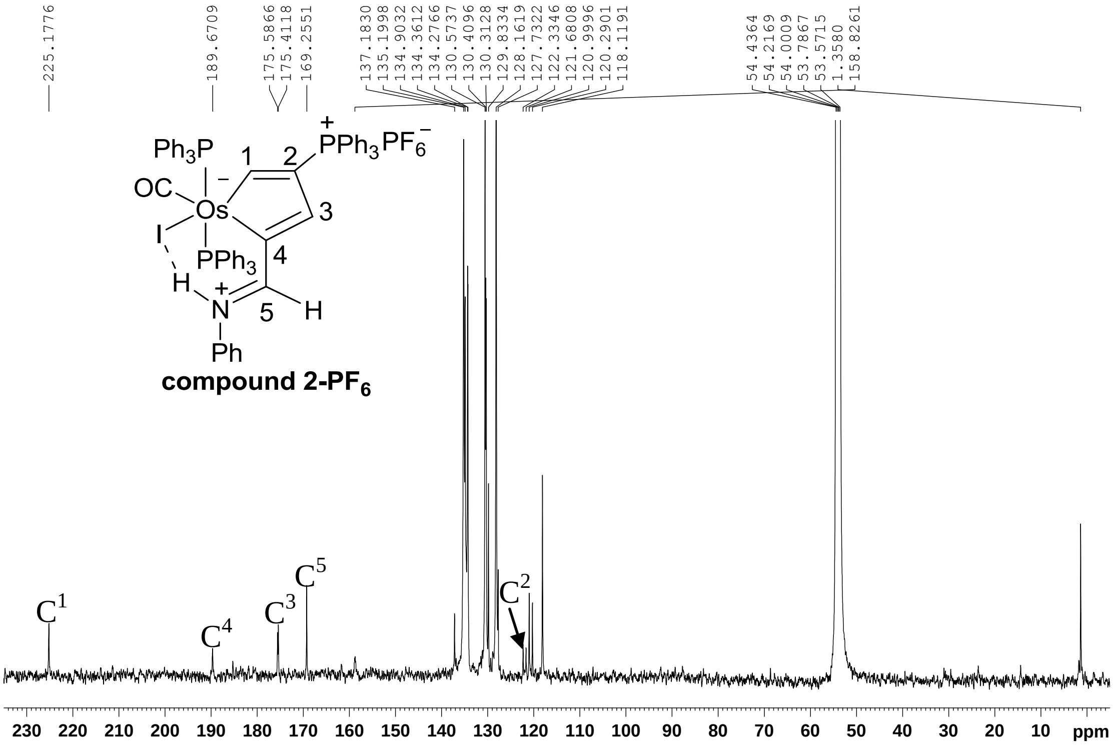

Figure S11. The $^{13}C\{^{1}H\}$ NMR (125.8 MHz, CD_2Cl_2) Spectrum for compound **2-PF$_6$** at room temperature.

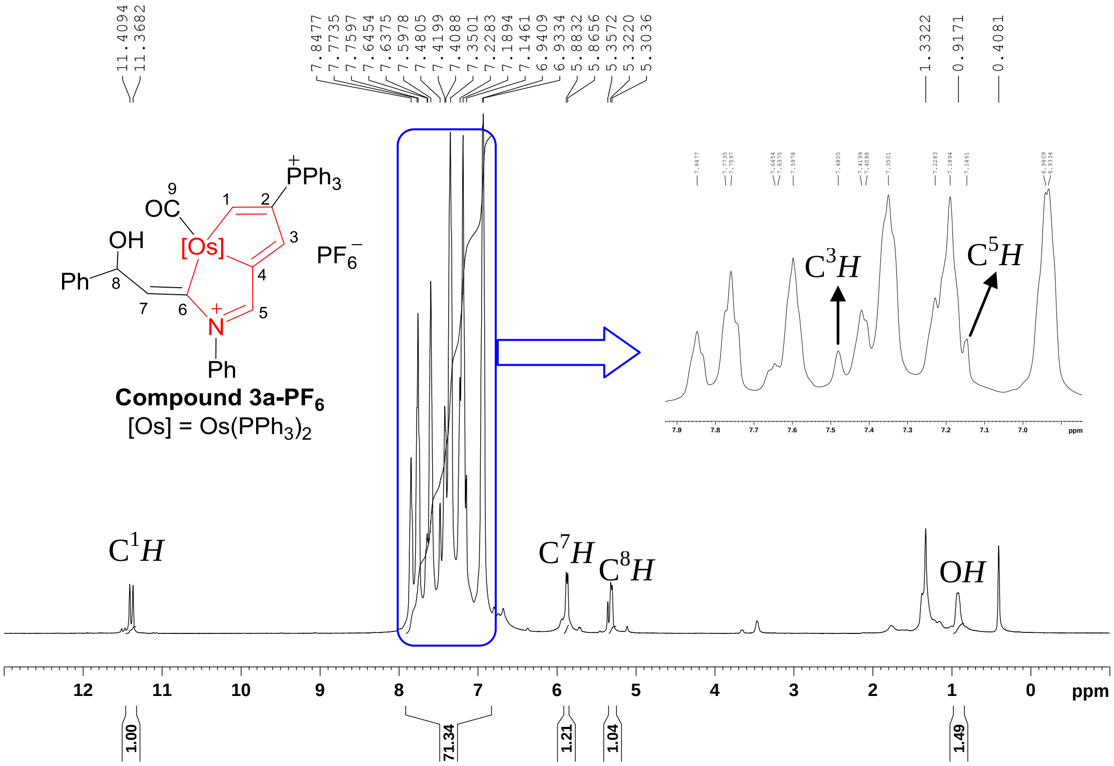

Figure S12. The ^{1}H NMR (500.2 MHz, CD_2Cl_2) Spectrum for compound **3a-PF$_6$** at room temperature.

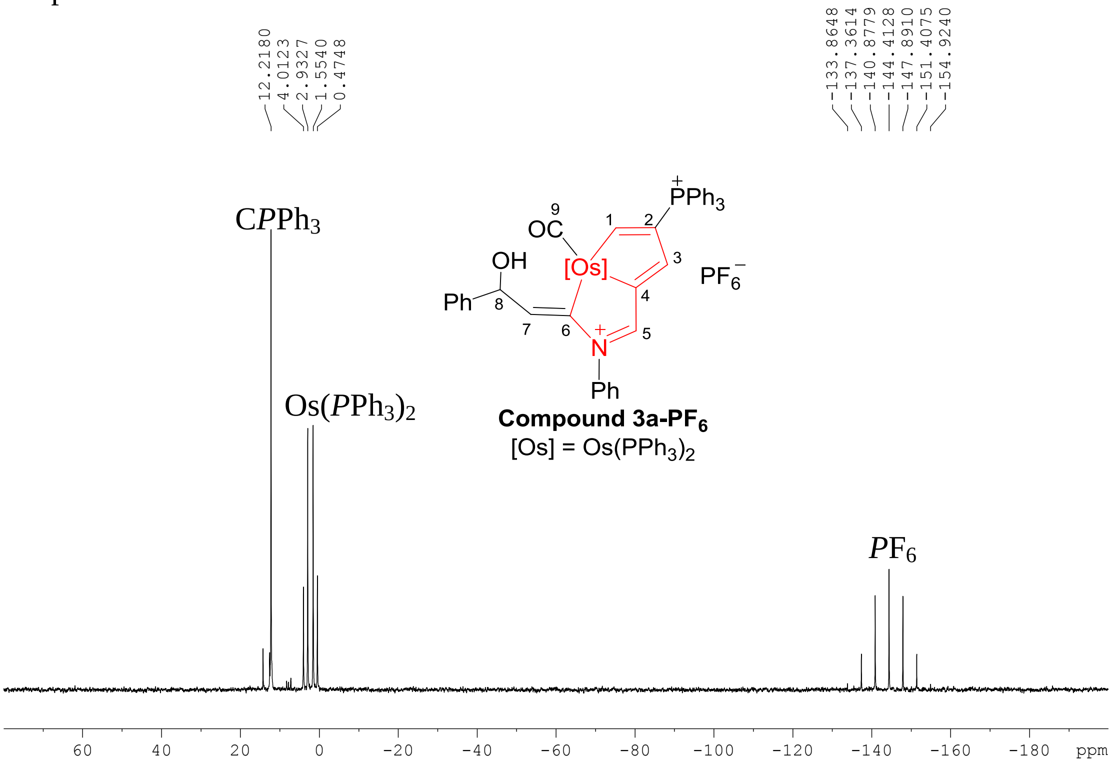

Figure S13. The ^{31}P{^{1}H} NMR (202.5 MHz, CD_2Cl_2) Spectrum for compound **3a-PF$_6$** at room temperature.

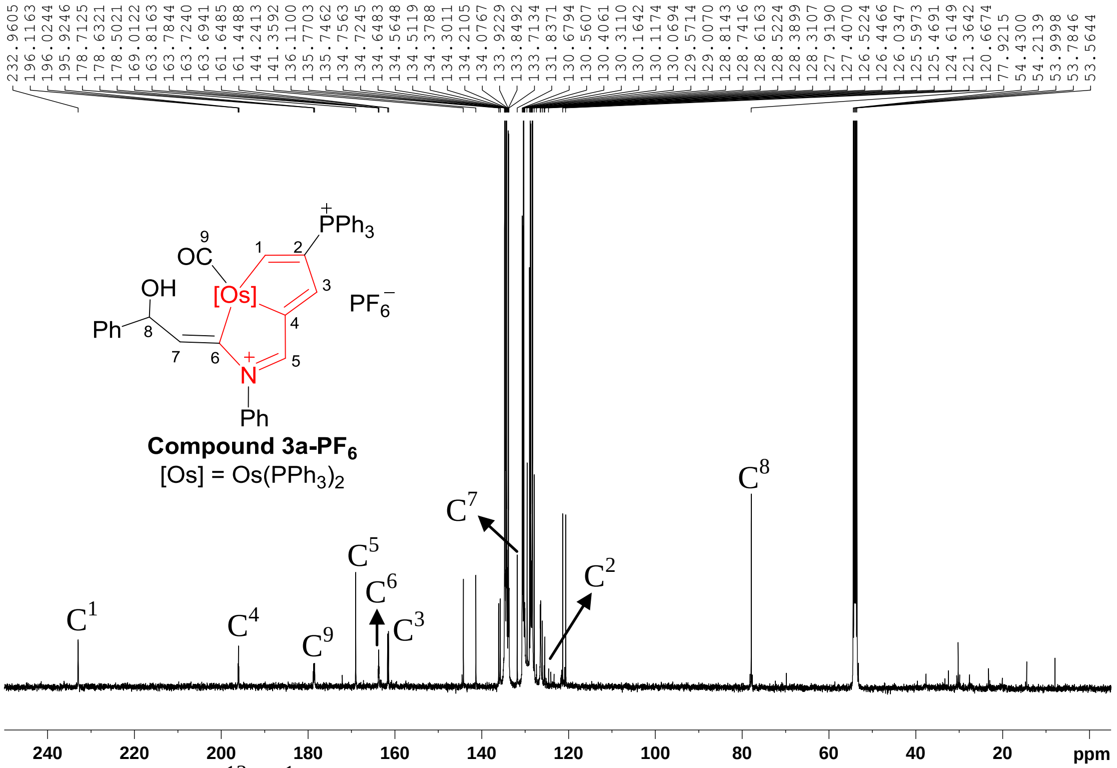

Figure S14. The ^{13}C{^{1}H} NMR (125.8 MHz, CD_2Cl_2) Spectrum for compound **3a-PF$_6$** at room temperature.

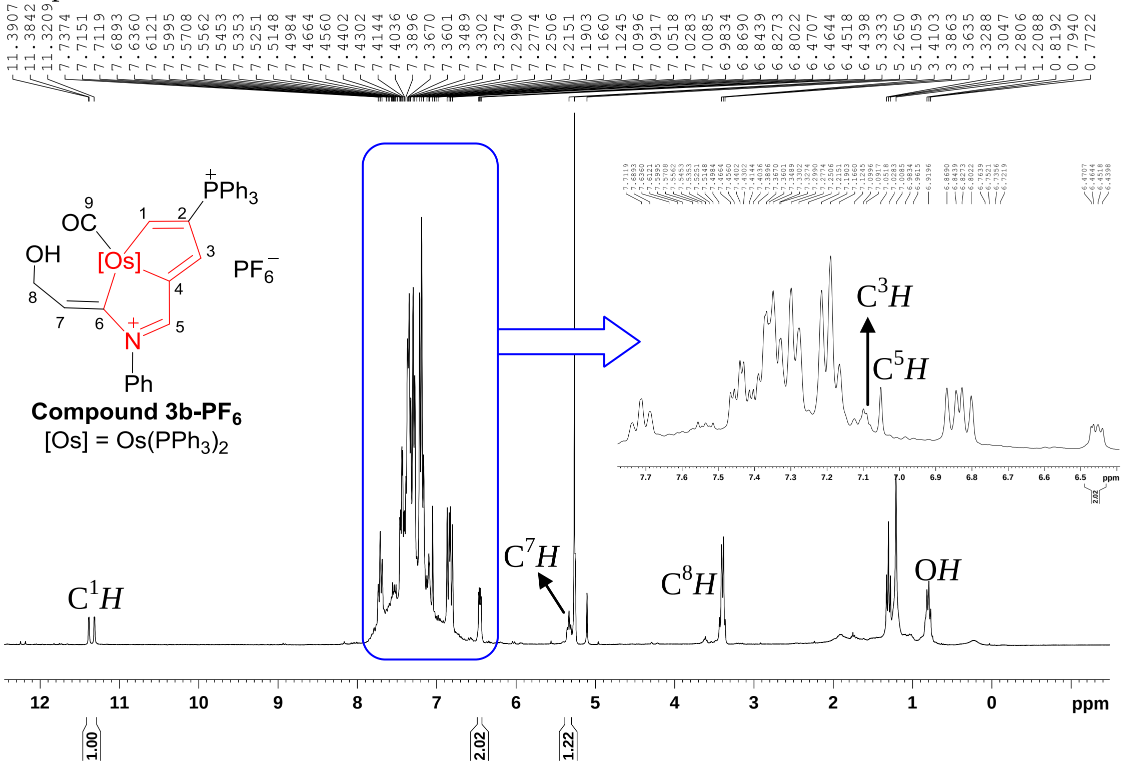

Figure S15. The ^{1}H NMR (300.1 MHz, CD_2Cl_2) Spectrum for compound **3b-PF$_6$** at room temperature.

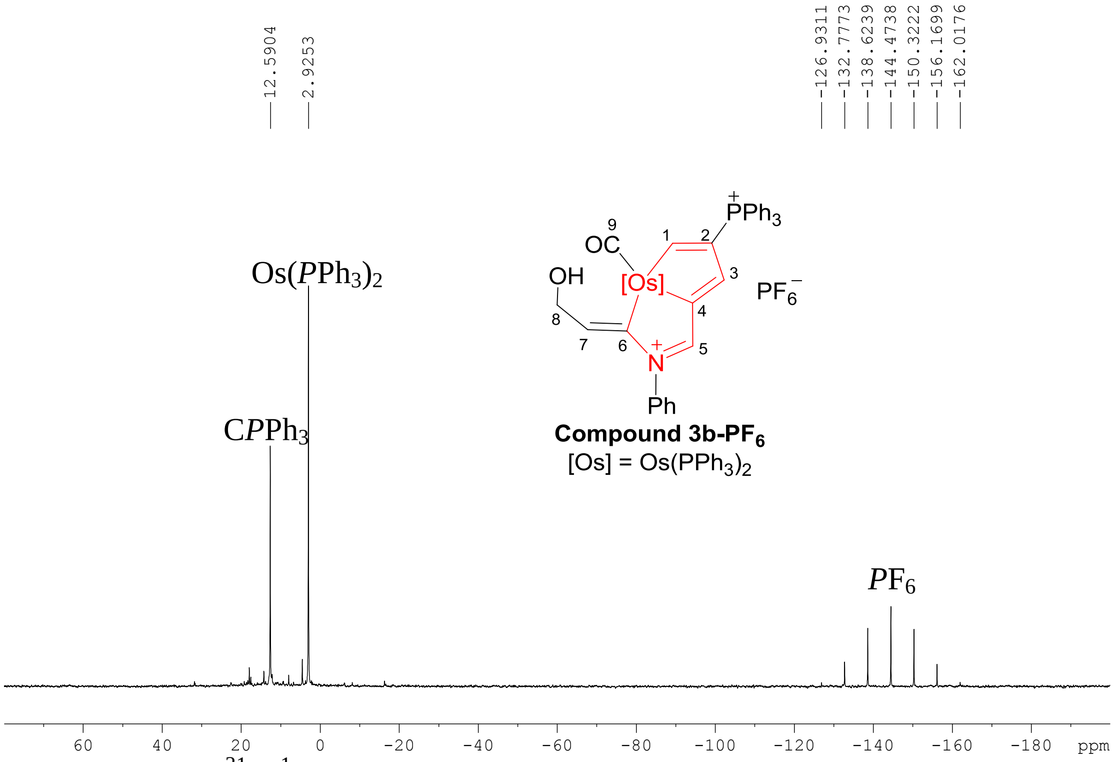

Figure S16. The ^{31}P{^{1}H} NMR (121.5 MHz, CD_2Cl_2) Spectrum for compound **3b-PF$_6$** at room temperature.

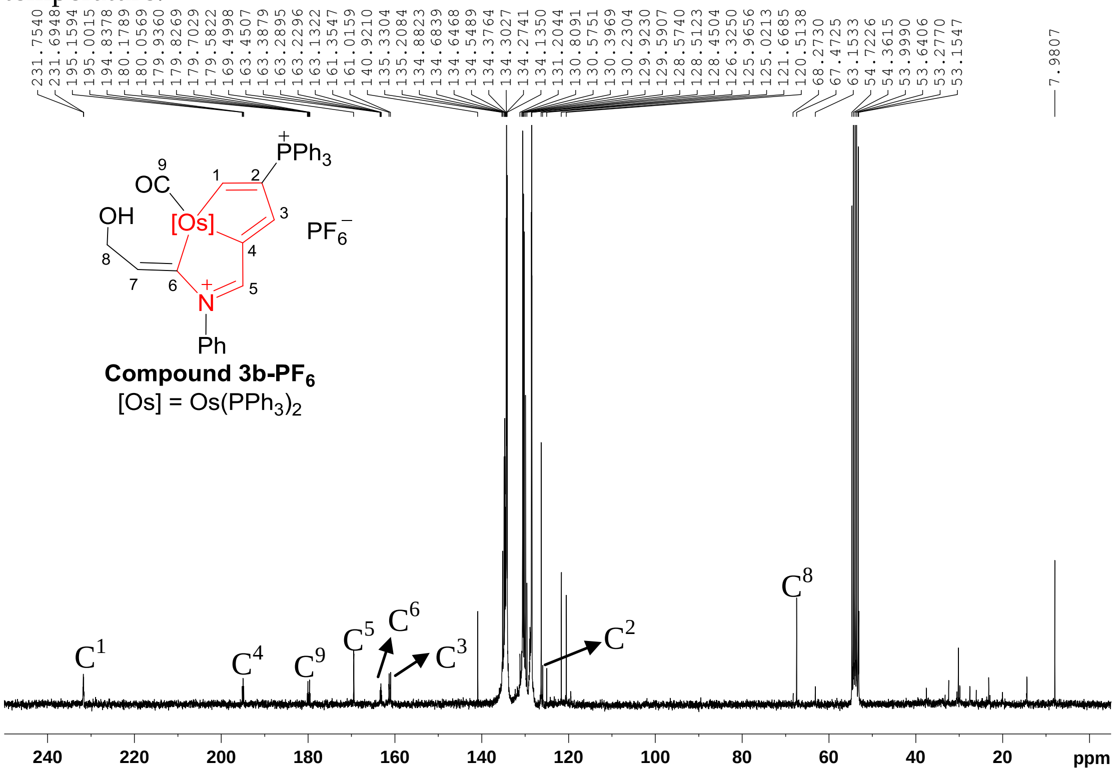

Figure S17. The ^{13}C{^{1}H} NMR (75.5 MHz, CD_2Cl_2) Spectrum for compound **3b-PF$_6$** at room temperature.

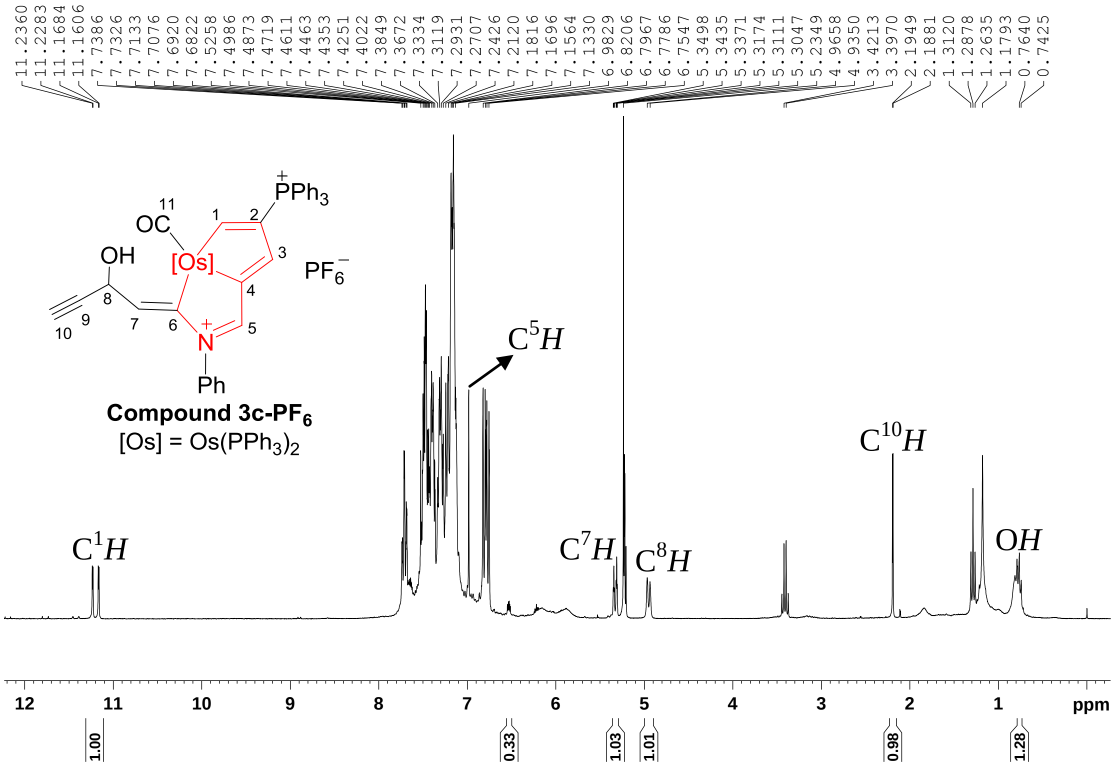

Figure S18. The ^{1}H NMR (300.1 MHz, CD_2Cl_2) Spectrum for compound **3c-PF$_6$** at room temperature.

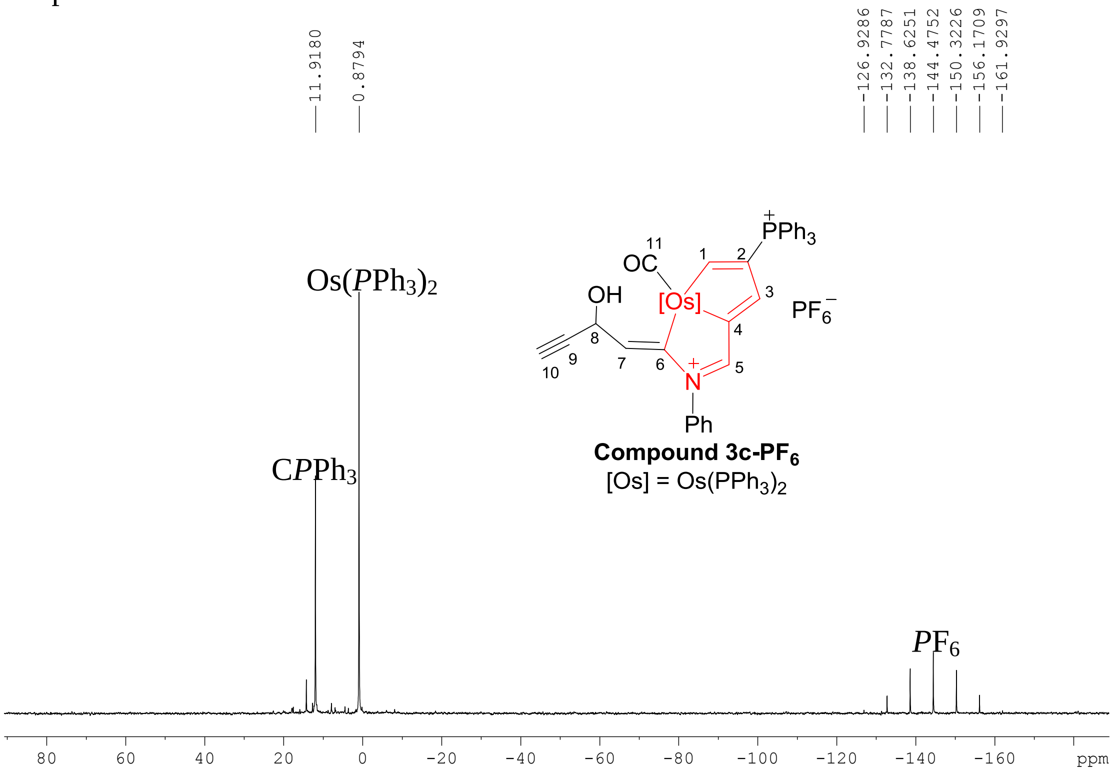

Figure S19. The $^{31}P\{^{1}H\}$ NMR (121.5 MHz, CD_2Cl_2) Spectrum for compound **3c-PF$_6$** at room temperature.

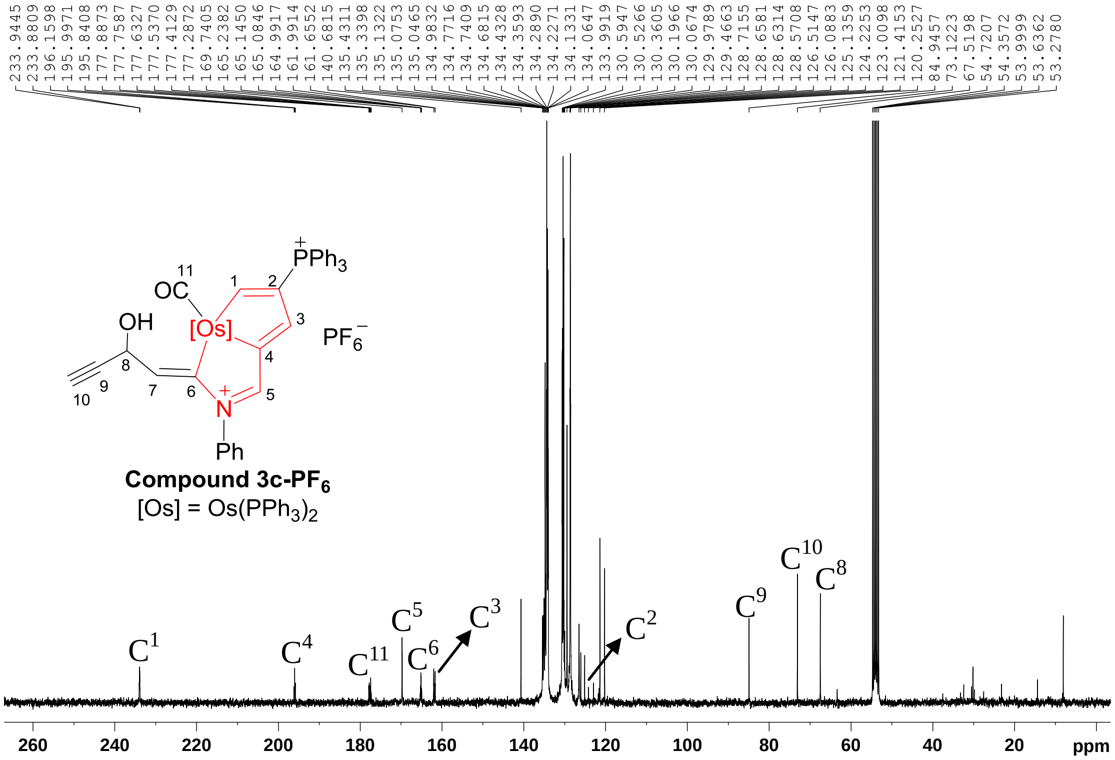

Figure S20. The $^{13}C\{^{1}H\}$ NMR (75.5 MHz, CD_2Cl_2) Spectrum for compound **3c-PF$_6$** at room temperature.

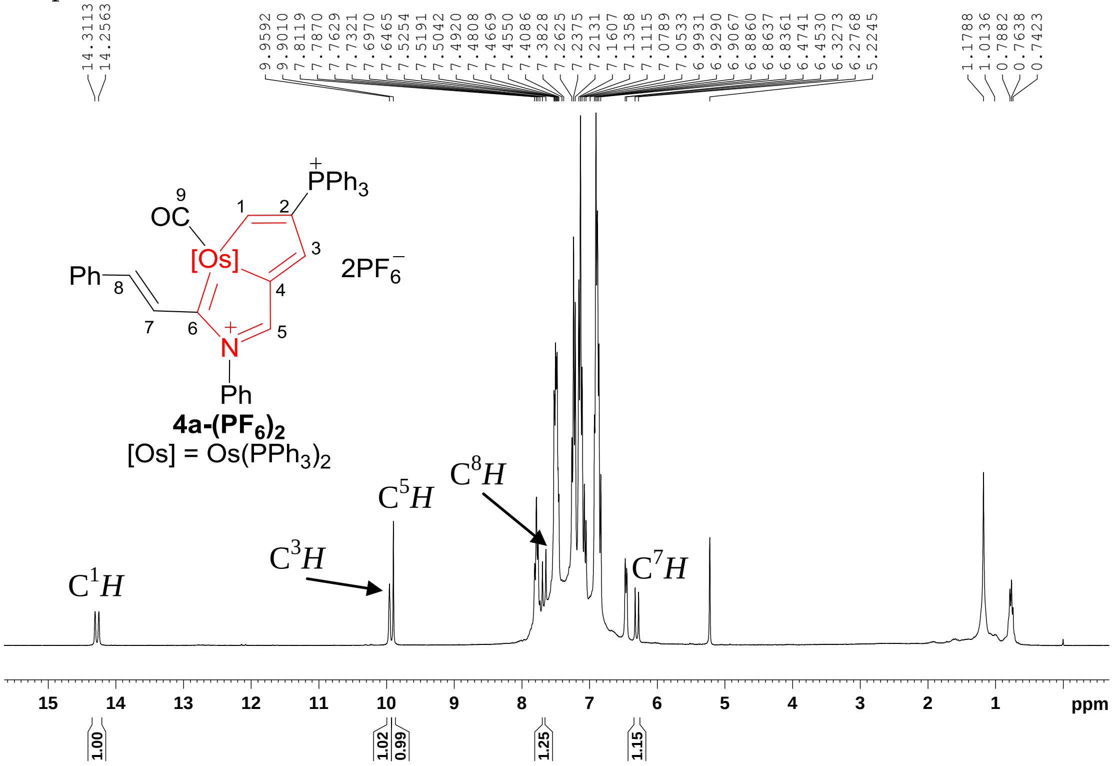

Figure S21. The ^{1}H NMR (300.1 MHz, CD_2Cl_2) Spectrum for compound **4a-(PF$_6$)$_2$** at room temperature.

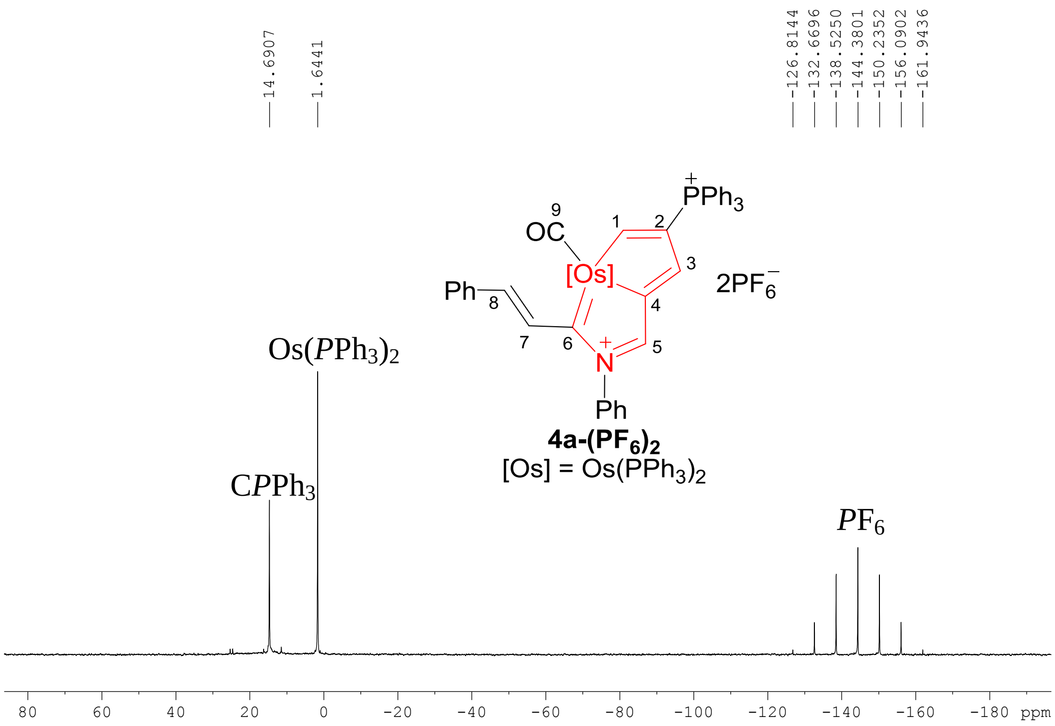

Figure S22. The $^{31}P\{^1H\}$ NMR (121.5 MHz, CD_2Cl_2) Spectrum for compound **4a-$(PF_6)_2$** at room temperature.

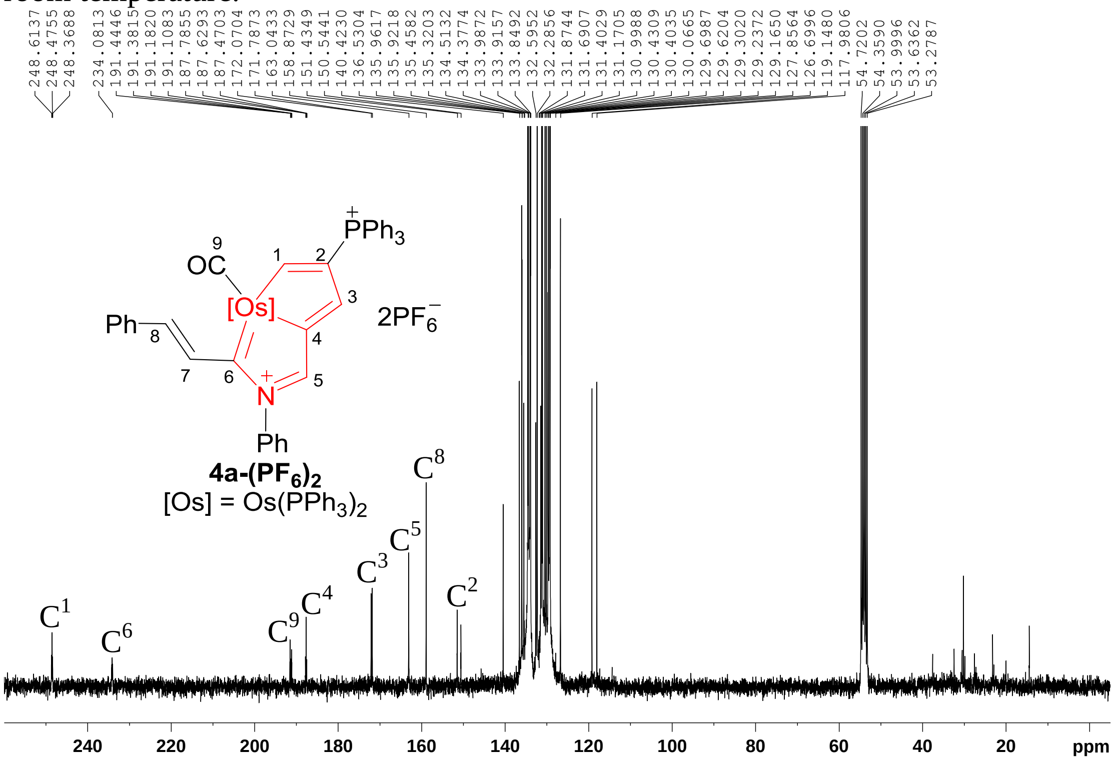

Figure S23. The $^{13}C\{^1H\}$ NMR (75.5 MHz, CD_2Cl_2) Spectrum for compound **4a-$(PF_6)_2$** at room temperature.

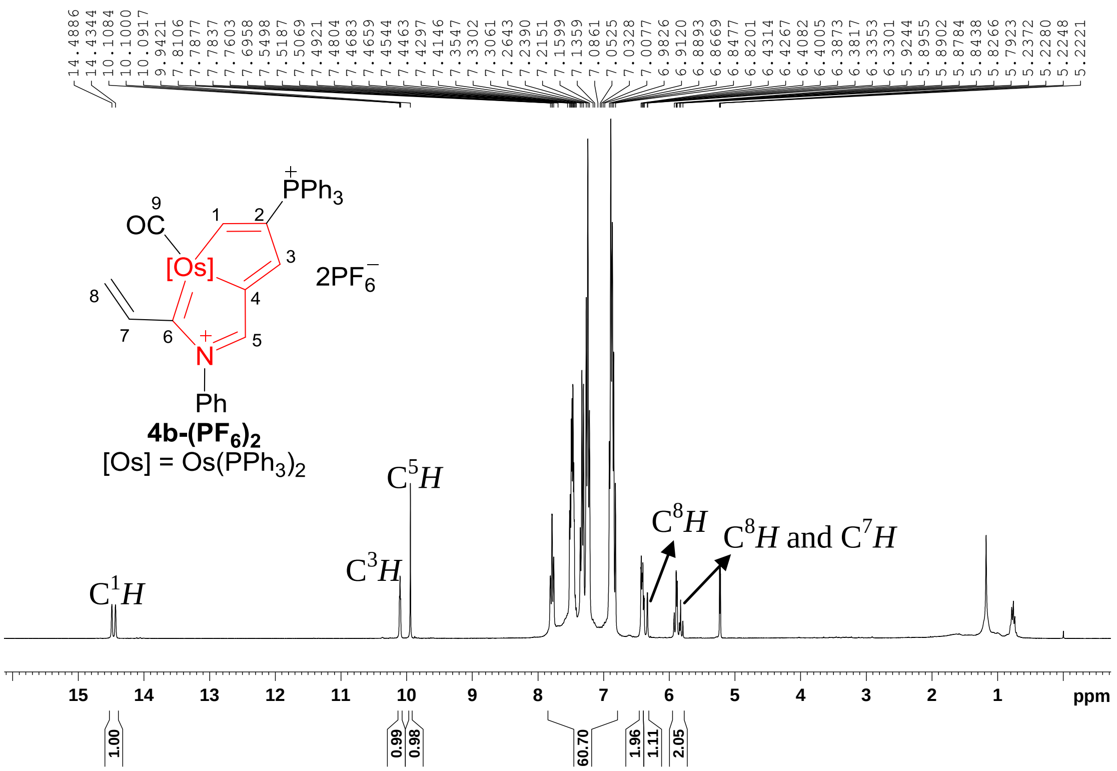

Figure S24. The ^{1}H NMR (300.1 MHz, CD_2Cl_2) Spectrum for compound **4b-(PF$_6$)$_2$** at room temperature.

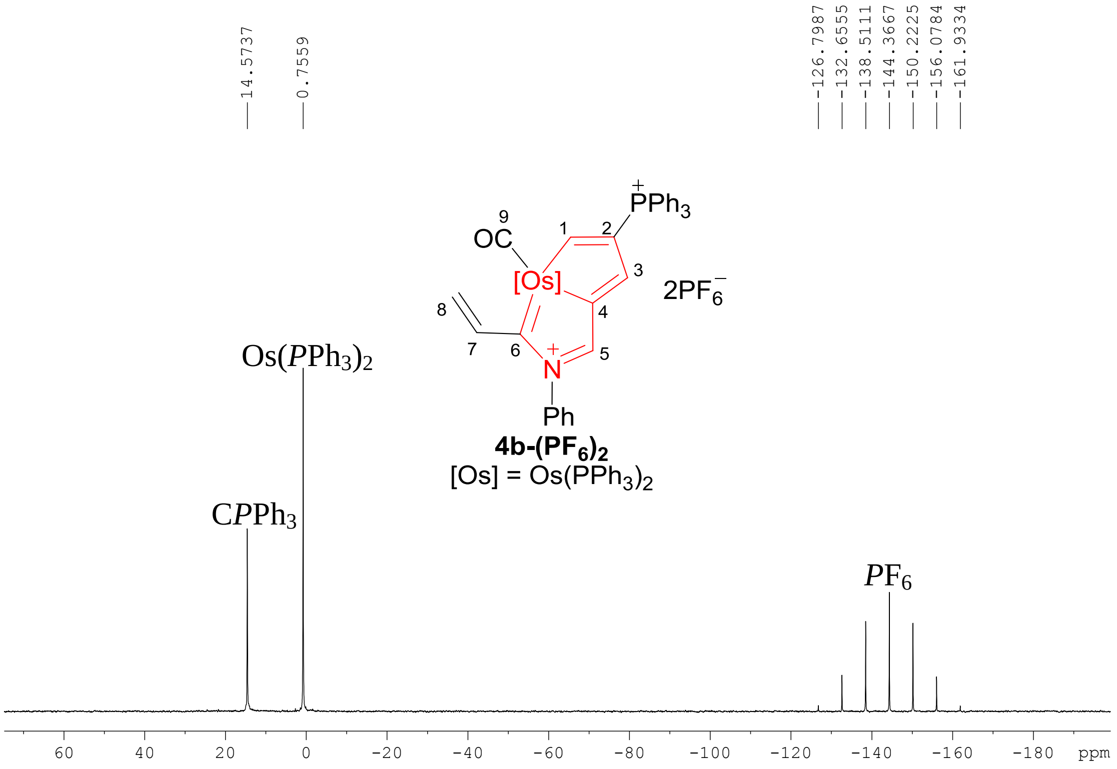

Figure S25. The ^{31}P{^{1}H} NMR (121.5 MHz, CD_2Cl_2) Spectrum for compound **4b-(PF$_6$)$_2$** at room temperature.

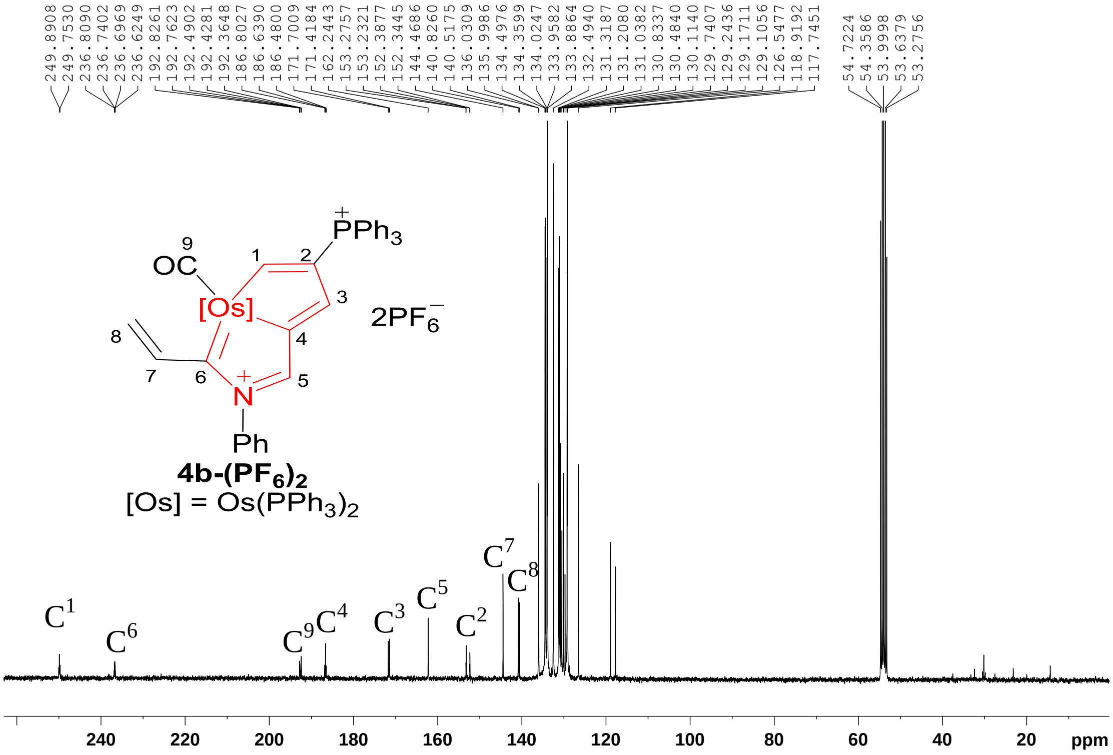

Figure S26. The $^{13}C\{^{1}H\}$ NMR (75.5 MHz, CD_2Cl_2) Spectrum for compound **4b-(PF$_6$)$_2$** at room temperature.

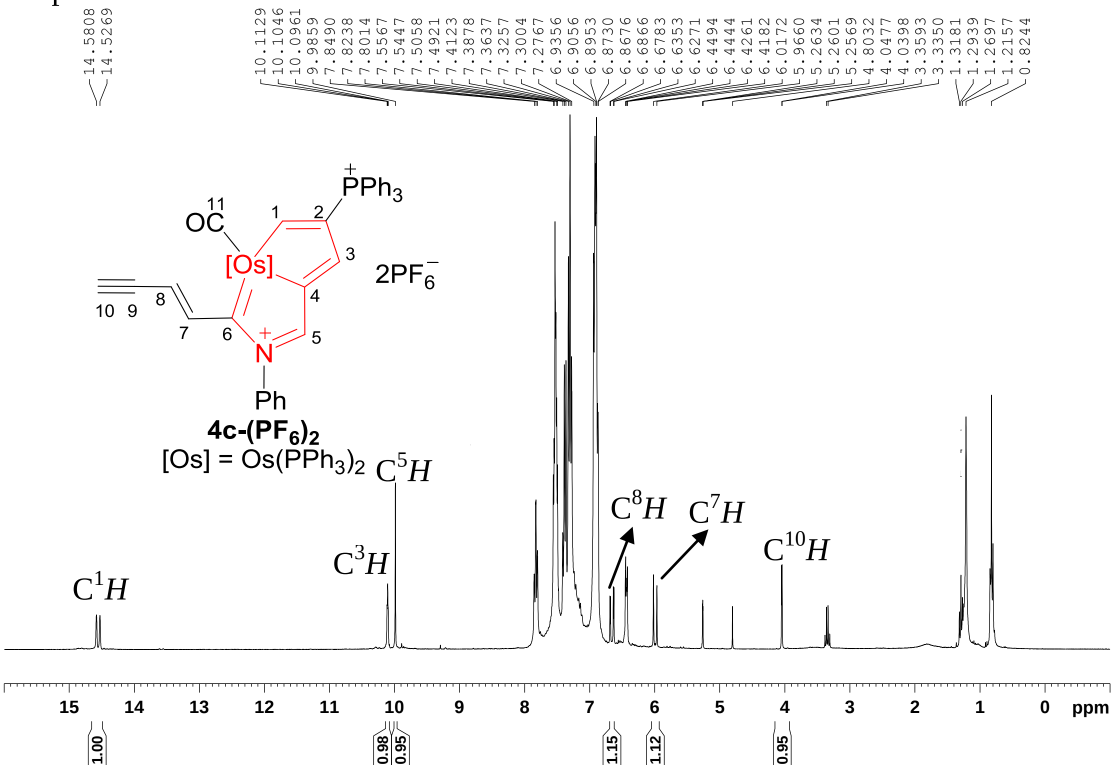

Figure S27. The ^{1}H NMR (300.1 MHz, CD_2Cl_2) Spectrum for compound **4c-(PF$_6$)$_2$** at room temperature.

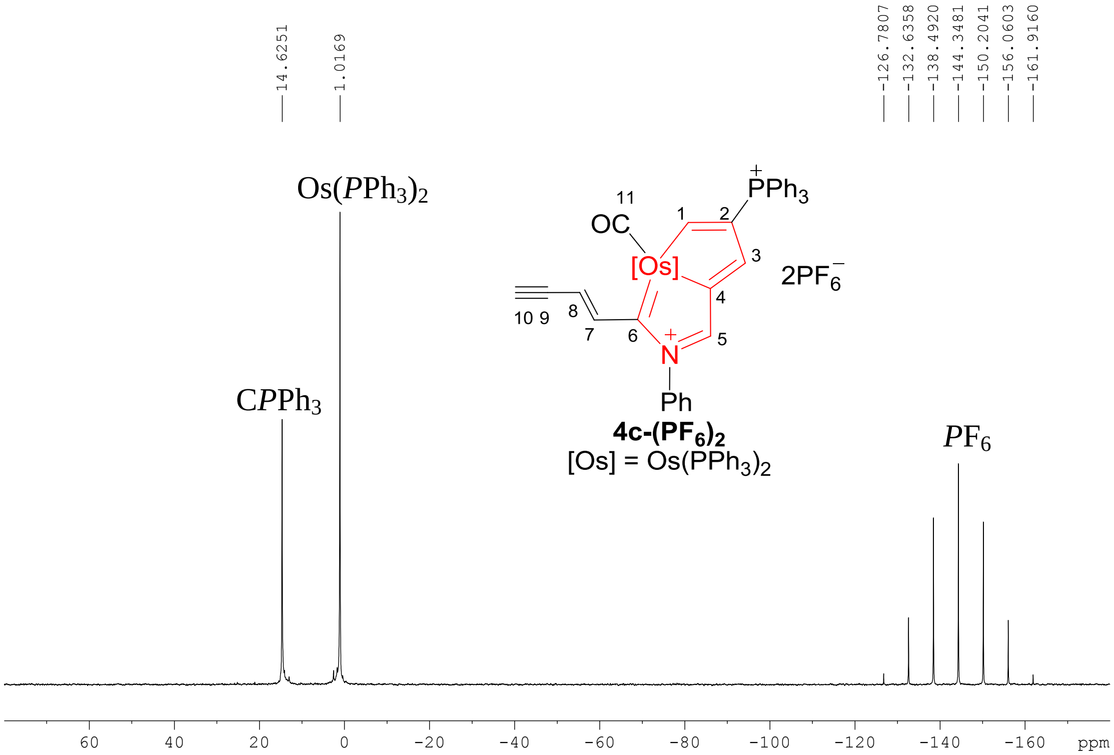

Figure S28. The ^{31}P{^{1}H} NMR (121.5 MHz, CD_2Cl_2) Spectrum for compound **4c-$(PF_6)_2$** at room temperature.

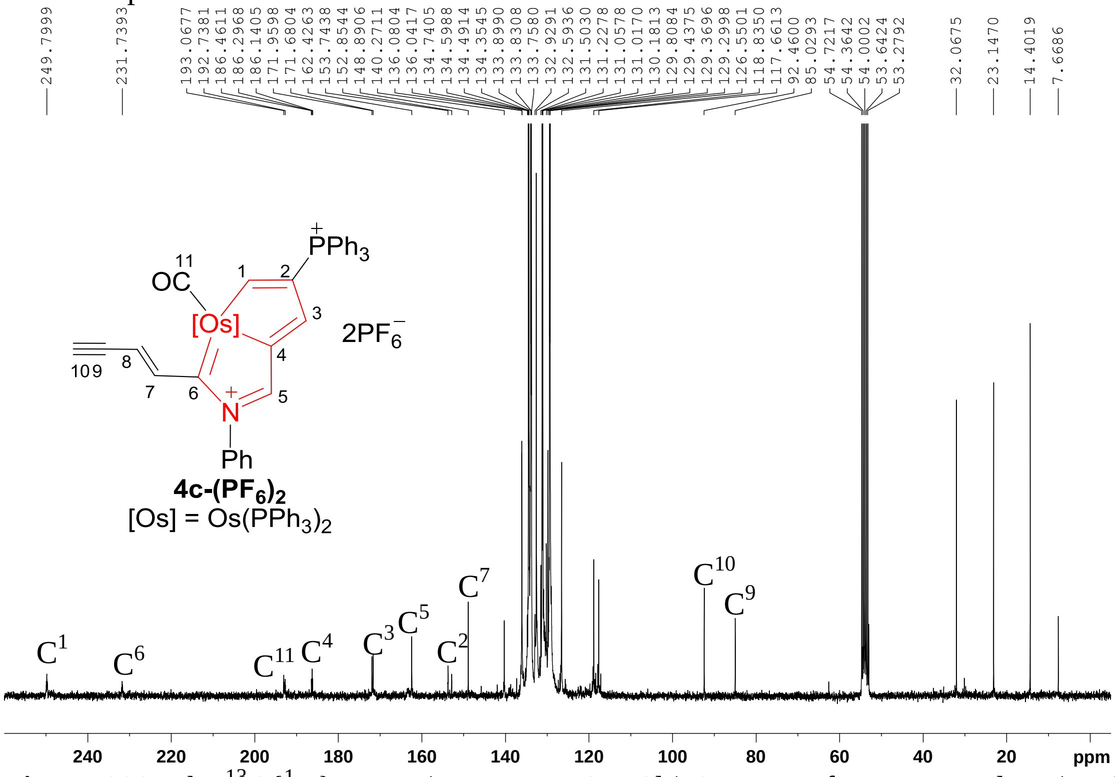

Figure S29. The ^{13}C{^{1}H} NMR (75.5 MHz, CD_2Cl_2) Spectrum for compound **4c-$(PF_6)_2$** at room temperature.

6. References

1. Xia, H. *et al.* Osmabenzenes from the Reactions of HC≡CCH(OH)C≡CH with $OsX_2(PPh_3)_3$ (X = Cl, Br) *J. Am. Chem. Soc.* **126,** 6862–6863 (2004).
2. Becke, A. D. Density-functional thermochemistry. III. The role of exact exchange. *J. Chem. Phys.* **98**, 5648–5652 (1993).
3. Miehlich, B., Savin, A., Stoll, H. & Preuss, H. Results obtained with the correlation energy density functionals of Becke and Lee, Yang and Parr. *Chem. Phys. Lett.* **157**, 200–206 (1989).
4. Lee, C., Yang, W. & Parr, R. G. Development of the Colle-Salvetti correlation-energy formula into a functional of the electron density. *Phys. Rev. B.* **37**, 785–789 (1988).
5. Hay, P. J. & Wadt, W. R. Ab initio effective core potentials for molecular calculations. Potentials for potassium to gold including the outermost core orbitals. *J. Chem. Phys.* **82**, 299–310 (1985).
6. Huzinaga, S. *Gaussian Basis Sets for Molecular Calculations* (Elsevier, Amsterdam, 1984).
7. Frisch M. J. *et al. Gaussian 03***,** *Revision E.01* (Gaussian, Inc., Wallingford, CT, 2004).
